# Discovery of multitargeting single agents as a novel route to the potential treatment of neurodegenerative diseases

**DOI:** 10.1016/j.bmcl.2026.130536

**Published:** 2026-01-07

**Authors:** Jeetal Vyas, Anuj S. Jamenis, Krishna Kaku, Yesha Shah, Kristin M. Miner, Tarun N. Bhatia, Roxanne E. Kim, Ruoli Bai, Ernest Hamel, Rehana K. Leak, Aleem Gangjee

**Affiliations:** a Division of Pharmaceutical Sciences, Duquesne University, Pittsburgh, PA, United States; b Molecular Pharmacology Branch, Developmental Therapeutics Program, Division of Cancer Treatment and Diagnosis, Frederick National Laboratory for Cancer Research, National Cancer Institute, National Institutes of Health, Frederick, MD 21702, United States

**Keywords:** Neurodegenerative diseases, Microtubule stabilization, Kinases, α-Synuclein

## Abstract

There are no cures for neurodegenerative diseases. The biggest hurdle to treating these disorders is that their clinical manifestation is rooted in multiple physiological processes. Therefore, efficacious pharmaceutical options will likely require two or more agents with different mechanisms of action. However, drug combinations have significant drawbacks, including overlapping toxicities and unique pharmacokinetic properties, particularly the rate and extent of central nervous system (CNS) penetration. A single agent with multiple mechanisms of action could overcome these drawbacks. We have recently discovered first-in-class novel single agents (compounds **1** and **2**) that mildly inhibit clinically important kinases and subtly favor microtubule stability at concentrations that show no evidence of neuronal toxicity in primary neurons, while maintaining their ability to penetrate the CNS *in vivo*. It is important to note that the effects of these analogs are mild and are predicated on avoiding neurotoxicity. These multitargeting single agents provide a new structural modality with the potential to influence treatments for Parkinson’s and Alzheimer’s disease and serve as lead compounds for further optimization.

## Introduction

Neurodegenerative diseases (NDs) such as Alzheimer’s disease (AD) and Parkinson’s disease (PD) have no cure.^1,2^ These diseases progressively damage neurons, leading to debilitating symptoms, such as motor dysfunction and cognitive decline.^1,2^ In the United States, AD affects over six million people^3^ and PD impacts nearly one million Americans.^4^ These numbers are expected to rise as the population ages.^3,4^ The increasing prevalence of these conditions creates an urgent need for innovative therapeutic approaches. NDs are multifactorial in nature and, therefore, single agents with unimodal targets have been unsuccessful as treatments. Thus, a multipronged approach may afford better treatment outcomes.^5^

The progression of NDs such as Lewy body disease (LBD) and AD are closely linked to the abnormal accumulation of hyperphosphorylated proteins, such as α-synuclein (α-syn) and tau.^6,7^ Tau is a microtubule-associated protein that stabilizes the structure of microtubules, which maintain cell shape and intracellular transport.^7^ Under physiological conditions, tau ensures efficient nutrient and cargo transport along axons by binding to and stabilizing microtubules.^7^ α-Syn plays a physiological role in synaptic vesicle trafficking.^7^ Under pathological conditions, these proteins become hyperphosphorylated and aggregated, contributing to neuronal dysfunction and cell death.^7,8^ In AD, hyperphosphorylated tau forms neurofibrillary tangles that can induce synaptic loss and neurodegeneration.^9^ The hyperphosphorylation of tau and α-syn has also been suggested as biomarkers of AD and LBD, respectively.^10^ Several kinases^11^ play a pivotal role in pathological hyperphosphorylation of α-syn and/or tau. Among those are Abelson non-receptor tyrosine kinase (ABL1),^12–14^ leucine-rich repeat kinase 2 (LRRK2),^15–21^ dual-specificity tyrosine phosphorylation-regulated kinase 1A (DYRK1A),^22–25^ and glycogen synthase kinase-3β (GSK3β).^26–30^ Mild targeting of these pathologically relevant kinases offers a potential therapeutic approach to slow or halt disease progression.^12–31^

In addition, mild targeting of microtubule dynamics also offers a promising approach to treating NDs.^32^ Microtubules are known to support neuronal structure, intracellular transport, and axonal growth.^32^ In diseases such as AD, the disruption of microtubule stability is primarily driven by the abnormal hyperphosphorylation of tau, possibly leading to the breakdown of microtubules and neuronal degeneration.^33^ Stabilizing microtubules or preventing their disassembly could protect neurons from the damaging effects of tau pathology.^33^ However, subtle modulation of microtubules is essential to avoid cytotoxicity,^34^ restore the structural integrity of neurons, and enhance axonal transport, potentially arresting neurodegeneration.^32,33,35^

NDs are the result of multifactorial processes and, therefore, the rationale for the use of two or more agents with different mechanisms of action is justifiable. However, multiple drug combinations have drawbacks, including overlapping toxicities and different pharmacokinetic properties, particularly CNS penetration, which can limit the safety and efficacy of combination therapies in NDs.^36^

**Fig. 1.**
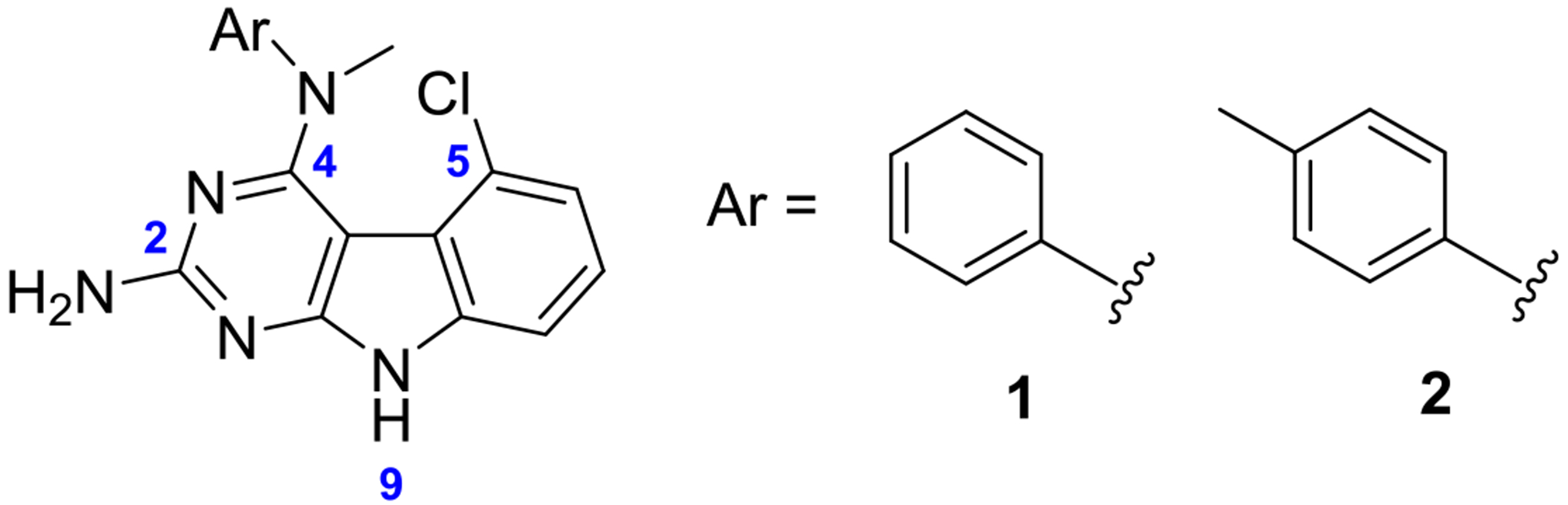
Structure of 1 and 2.

While multitargeted therapy has been speculated to have therapeutic potential against NDs, to our knowledge the combination of mild kinase inhibition and tubulin modulation has not been explored in a single agent.^37,38^ We have recently discovered first-in-class novel single agents **1** and **2** (Fig. 1) that mildly inhibit relevant kinases (Table S2) and also mildly modulate (stabilize) tubulin^39^ without neuronal toxicity. In addition, these compounds penetrate the CNS *in vivo*. After accumulating evidence that this novel multitargeted mechanism (kinase inhibition and tubulin modulation) in single agents may impact the accumulation of pathological α-syn and, by extension, tau, they may provide new modalities for the treatment of AD and LBD.

## Results and discussion

We previously synthesized compounds **1** and **2** as potential anticancer agents. We determined that these compounds were ineffective as anti-tumor agents due to mild effects on tubulin as well as a lack of tumor cell inhibition.^39^ This mild modulation (particularly mild stabilization) of tubulin without cytotoxicity suggested an evaluation in NDs that would benefit from tubulin stabilizing agents.^32,33,35^ Recently, on the basis of structural similarities of compounds **1** and **2** with known kinase inhibitors involved in phosphorylation of tau and α-syn,^40–43^ it became apparent that compounds **1** and **2** could function as multitargeted mild inhibitors of appropriate kinases as well as mild tubulin stabilization agents. Hence, we tested compounds **1** and **2** in pathologically relevant kinases and discovered mild multi-kinase inhibitory activity for four kinases (ABL1, DYRK1A, LRRK2, and GSK3β).

Prior to biologically evaluating compounds **1** and **2** further and designing potentially new analogs, it was necessary to carry out molecular modeling and *in silico*^44^ calculations to provide a rationale for their interactions with tubulin and the kinases (ABL1, DYRK1A, LRRK2, and GSK3β).

### Molecular modeling

**Fig. 2.**
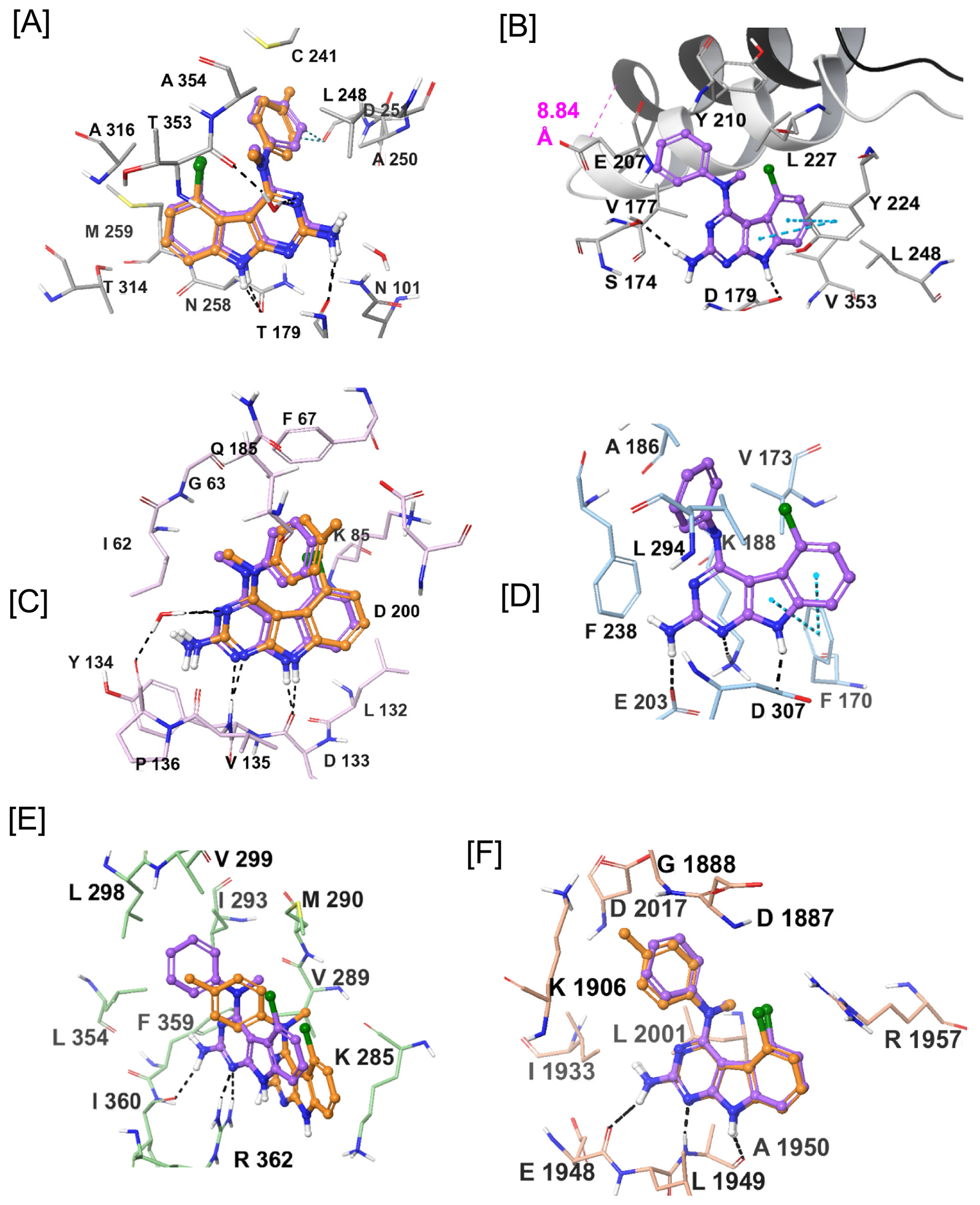
Docked poses of compounds **1** and **2** in different targets. (A) Docked pose of **1** (purple) and **2** (orange) in colchicine site of tubulin (PDB ID: 4O2B). (B) Docked pose of **1** (purple) in the vinca site of tubulin (PDB ID: 5NJH, gray) superimposed with the crystal structure of vinblastine (not shown) bound tubulin (PDB: 5J2T, black). (C) Docked pose of **1** (purple) and **2** (orange) in GSK3β (PDB ID: 7B6F). (D) Docked pose of compound **1** (purple) in the binding site of DYRK1A (PDB ID: 7OY6) (E) Docked pose of compound **1** (purple) and **2** (orange) in ABL1 (PDB ID: 3CS9). (F) Docked pose of compound **1** (purple) and **2** (orange) in LRRK2 binding site (PDB ID: 8U7H). (For interpretation of the references to colour in this figure legend, the reader is referred to the web version of this article.)

**Fig. 3.**
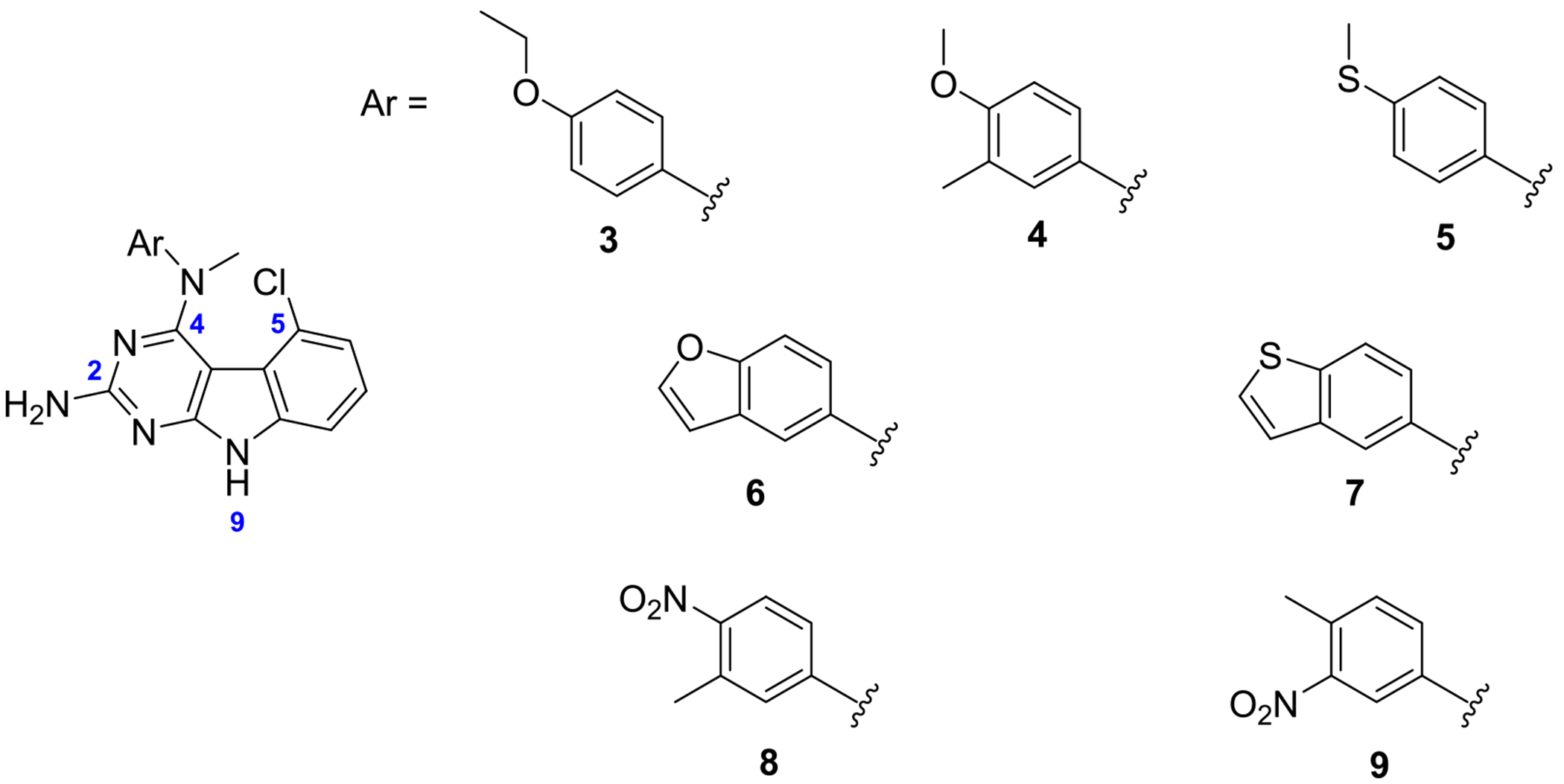
Structure of novel compounds **3–9** (Series I).

**Fig. 4.**
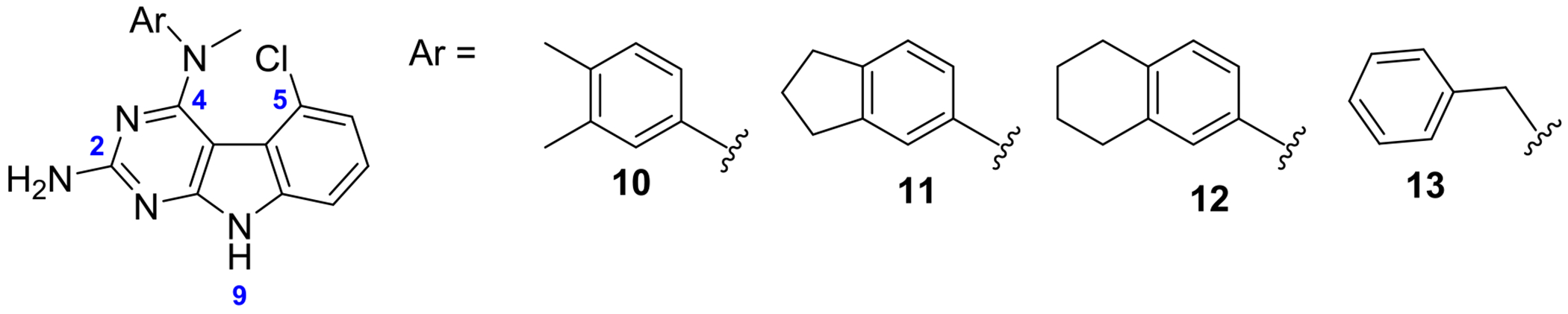
Structure of novel compounds **10–13** (Series II).

We initially reported, on the basis of *in vitro* data,^39^ that compounds **1** and **2** exert mild tubulin modulatory effects by poorly binding to the colchicine site on tubulin, compared to colchicine, and exerting a destabilizing effect. In the colchicine site (PDB ID: 4O2B),^45^ compounds **1** and **2** have similar docked poses (Fig. 2A), with the tricyclic scaffold of each involved in hydrogen bond interactions with Thr179, Thr353, and Asn258. The 9-NH and 2-NH_2_ are involved in hydrogen bond interactions with Asn258 and Thr179, while 3-N of the pyrimidine ring forms a water mediated hydrogen bond with Thr353. The *N^4^*-substituents fit snuggly into the ligand binding pocket and form aromatic hydrogen bonds with Asp251. Since the interactions at the colchicine site on tubulin leads to detrimental mild destabilization, it was important to increase the bulk on the *N^4^*-phenyl ring of compound **1** to provide steric hindrance, with the aim of decreasing binding to the colchicine site and, hence, the destabilization of tubulin. (See Figs. 3 and 4.)

To this end, we proposed compounds **3**–**9** (Series I) with additional bulky substituents at the *N^4^*-position with varying electronics. Compounds **3**–**5** explored an electron donating substituent on the para position of the *N^4^*-aromatic substituent. The design of compounds **6** and **7** involved conformationally restricting the alkoxy or thioalkyl substituents, respectively in a ring. Compounds **8** and **9** explored the presence of strong electron withdrawing groups on the *N^4^*-aromatic substituent.

In order to explore bulky alkyl substitutions (compound **10**) along with conformational rigidity (compounds **11**, **12**) as well as flexibility (compound **13**), we designed compounds **10**–**13** (Series II). Substituents like indane (compound **11**) and tetralin (compound **12**) impose rigid conformations, which contrasts with the flexibility offered by the benzyl group in compound **13**.

Upon docking in the colchicine site on tubulin, it was observed that, to accommodate the larger *N^4^*-substituent in Series I and II, the scaffold shifts and loses hydrogen bonding interactions with Thr179, Thr353 and Asn258 (representative compounds shown in Fig. S1A), which results in a poorer docked score. However, for polar substituents on the para position of the *N^4^*-phenyl substituent (compounds **3**, **4**, **6** and **8**), interaction with Cys241 led to a better docked score (Table S1). Comparing the docked scores (Table S1) of compounds **1**–**13** with colchicine at this site on tubulin, we observed that most of the proposed compounds were weakly bound compared to colchicine and were not expected to provide destabilizing effects on tubulin.

In our previous report,^39^ we speculated that the tubulin stabilization effect could be the result of binding at an alternate site on tubulin. To explore the potential stabilizing effects of compounds **1**–**13**, a docking study in the vinca site (PDB ID: 5NJH)^46^ (Fig. 2B) was prompted by a report^46^ of crystal structures of triazolo-pyrimidine compounds in the vinca site of tubulin. The 2-NH_2_ moiety of compound **1** affords a hydrogen bond with Ser174, while the tricyclic scaffold forms a π-π stacking interaction with Tyr224. Importantly, docked compound **1** caused a ~8.8 Å inward shift of the α-helix of αH10-tubulin (Fig. 2B, gray ribbon) compared to vinblastine-bound tubulin (Fig. 2B, black ribbon). This inward shift brings the α-helix closer to the β1-tubulin monomer resulting in a less-curved microtubule assembly, thus precluding microtubule disintegration and promoting mild microtubule stabilization.^46^ Vinca causes an opposite effect by creating a wedge in the microtubule assembly and pushing the helix outward that results in tubulin destabilization.^47^ The inward movement of the α-helix of αH10-tubulin has been previously reported to lead to tubulin stabilization.^46^ Interestingly, compound **2** also demonstrates this important inward shift of the α-helix of αH10-tubulin but by only ~7 Å this may vary its stabilizing effects on tubulin compared to compound **1** (Fig. S1B). The modeling study of compound **2** indicates a loss of the π-π stacking interaction between the tricyclic scaffold and Tyr224 (compared to compound **1**) and could account for the lower shift in the α-helix for compound **2**.

Compounds **3**–**13** were designed to potentially hinder the colchicine site binding and to utilize the aromatic bulk to form additional interactions in the vinca site, which would promote further tubulin stabilizing effects. The *N^4^*-substituent of compounds **3**–**13** are able to afford additional hydrophobic interactions. This results in better docked scores for compounds **3–13**, in the vinca site, compared to compounds **1** and **2** (Table S1). However, the inward shift of the α-helix ranges from 7 to 8 Å indicating that the stabilizing effects at vinca site may not be as pronounced as they are for compound **1**. It is important to note that mild tubulin stabilization is the goal.

As mentioned above, on the basis of the structural similarities of compounds **1**–**13** to known kinase inhibitors^40–43^ it was of interest to determine the docked poses and scores of compounds **1**–**13** in kinases known to be involved in tau and α-syn phosphorylation.^12–31^ We performed docking studies in all relevant kinases^11^ implicated in the pathology of NDs and in particular in DYRK1A (PDB ID: 7OY6),^48^ GSK3β (PDB ID: 7B6F),^40^ LRRK2 (PDB ID: 8U7H),^49^ and ABL1 (PDB ID: 3CS9)^50^ as compounds **1** and **2** were found to be inhibitors of these four kinases.

Compounds **1** and **2** form multiple hydrogen bond interactions in the ATP pocket of GS3Kβ (PDB ID: 7B6F).^40^ The 9-NH, 1-N, and 3-N form hydrogen bond interactions with Asp133, Val135 and Pro136 (Fig. 2C). The 1-N forms a water-mediated hydrogen bond with Pro136. Similar interactions were observed for compounds from Series I and II (representative compounds shown in Fig. S1C) with the *N^4^*-substituent forming additional π- π stacking interaction with Phe67. Compound **1** docks in the DYRK1A (PDB ID: 7OY6)^48^ (Fig. 2D) ATP site with the pyrimido [4,5-*b*]indole scaffold engaged in hydrogen bond interactions with Asp307, Glu203 and Lys188. Phe170 forms π- π stacking interactions with the scaffold of compound **1**. The docked poses and docked scores for compounds **2**–**13** are comparable to compound **1** (Table S1 and representative compounds shown in Fig. S1D).

Compounds **1** and **2** docked similarly in the ATP site of ABL1 (PDB ID: 3CS9)^50^ (Fig. 2E) and had similar interactions with the kinase. Hydrogen bond interactions were observed between 1-N and 2-NH_2_ and Arg362 and Ile360, respectively. In addition to these interactions, the scaffolds of compounds **6** and **12** were involved in cation-π interactions with Lys285 (Fig. S1E) while compounds **3**–**5**, **7**–**11** and **13** had similar docked poses and scores compared to **1** and **2**. Docked compounds **1** and **2** in the ATP site of LRRK2 (PDB ID: 8U7H)^49^ involve three hydrogen bond interactions with Ala1950 and Glu1948 (Fig. 2F), with compounds **3**–**13** displaying similar docked poses and docked scores (Table S1 and representative compounds shown in Fig. S1F).

On the basis of our molecular modeling study, the mild tubulin modulatory activity as well as the docked scores for mild appropriate kinase inhibitory activities of compounds **1**–**13** afforded a rationale for biological evaluations.

### Syntheses

**Scheme 1.**
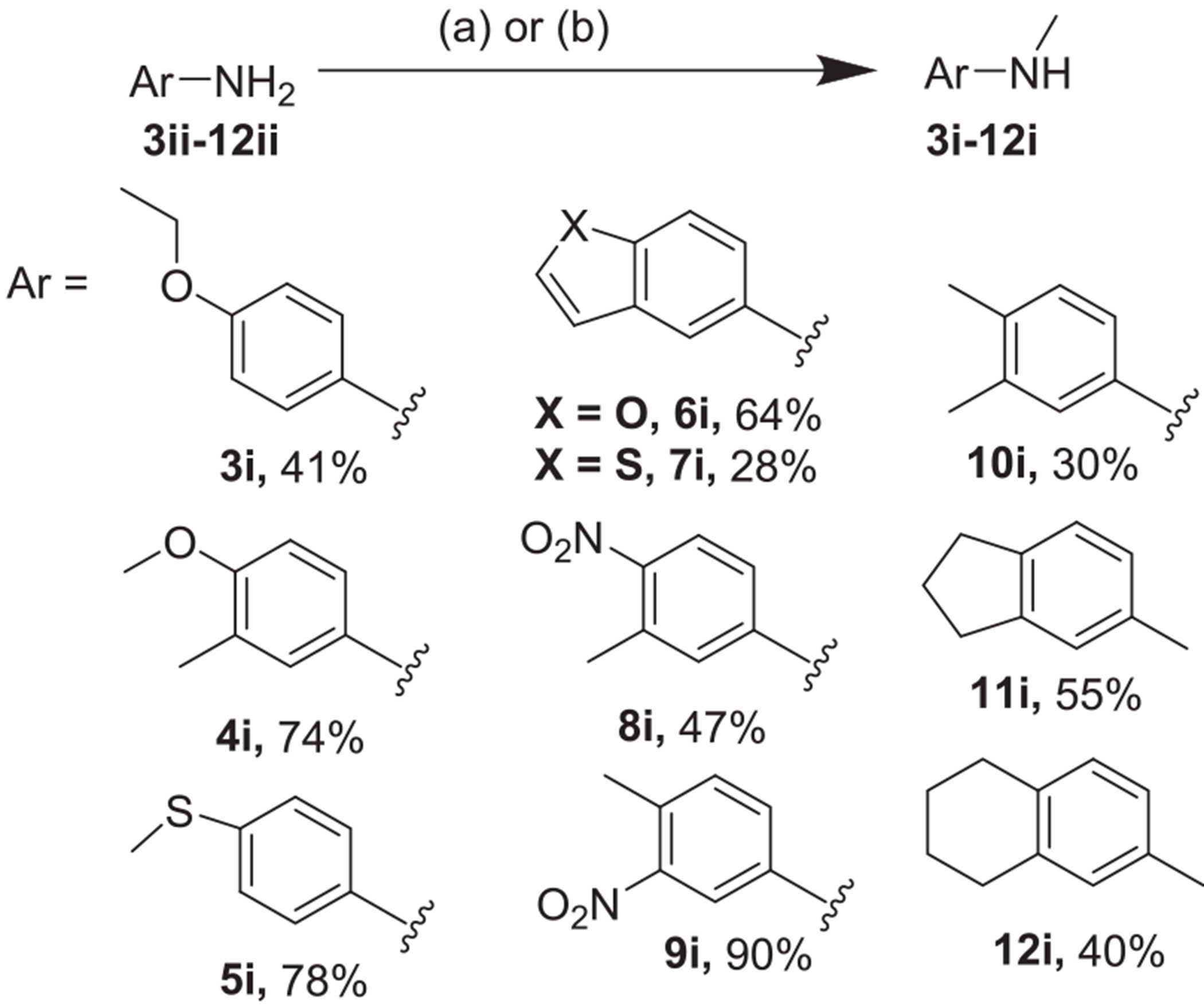
^a^. ^a^ Reagents and conditions (a) (i) **3ii**, **4ii, 6ii-12ii**, NaOMe, MeOH, paraformaldehyde, rt., overnight. (ii) sodium borohydride, reflux, 2–5 h. (b) **5ii**, MeI, THF, 0–5 °C, 3 h.

*N*-methylation utilizing methyl iodide and reductive amination using paraformaldehyde and the appropriate primary amines (compounds **3ii**-**12ii**) (Scheme 1) afforded secondary amines (compounds **3i**–**12i**).

**Scheme 2.**
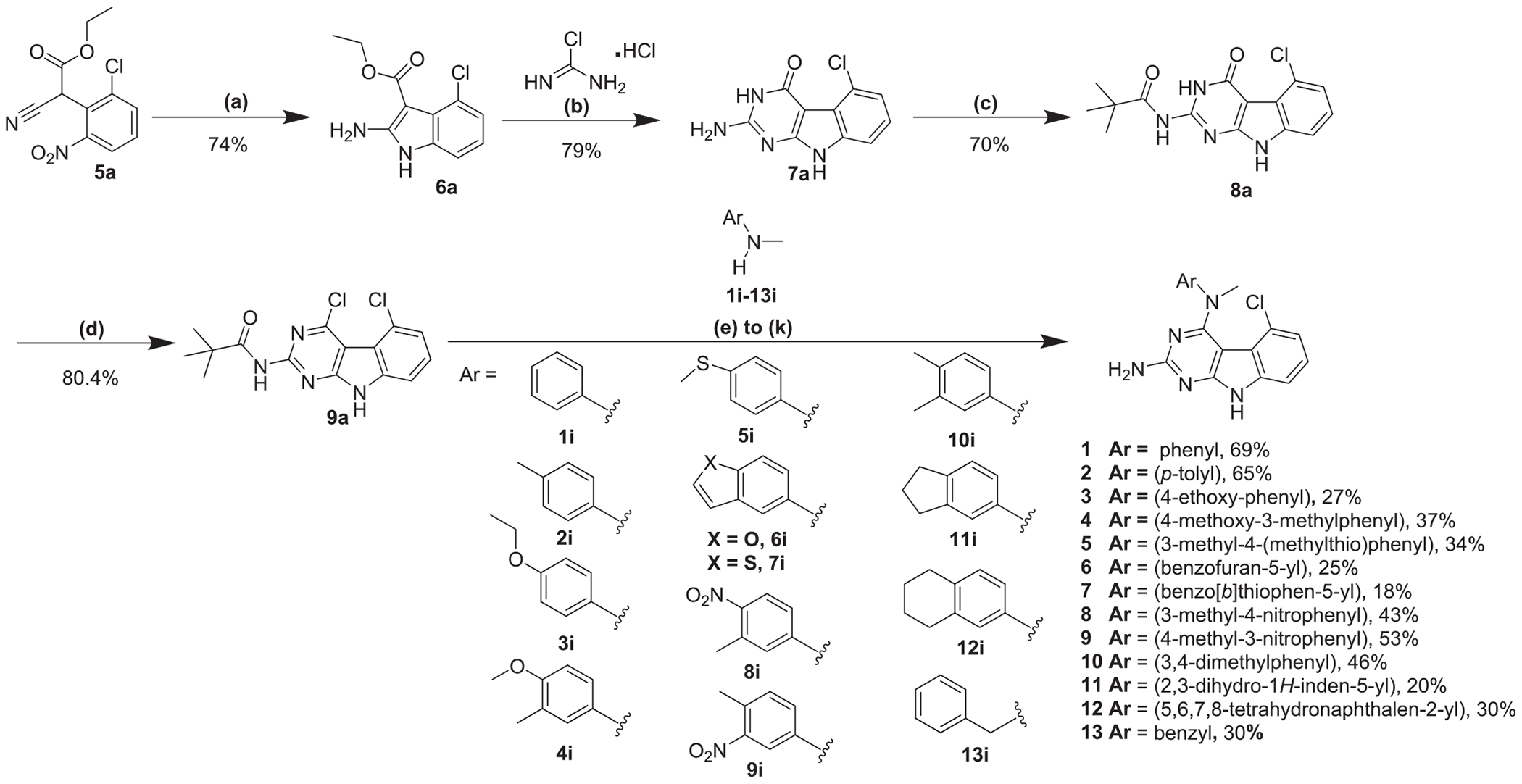
^b^. ^b^ Reagents and conditions (*a*) Zn dust, acetic acid, 70 °C, 6 h (*b*) Methyl sulfone, 120 °C, 4 h (*c*) Piv_2_O, 120 °C, 3 h (*d*) POCl_3_, 110 °C, 4 h (*e*) (i) **1i**, **2i**, **8i**, **11i**, or **13i**, acetonitrile, HCl (cat.), microwave 80 °C, 8 h (ii) 1 N NaOH, reflux, 5 h (*f*) (i) **6i** or **7i**, n-BuOH, HCl (cat.), microwave, 150 °C, 4 h (ii) 1 N NaOH, reflux, 4 h (*g*) (i) **3i** or **12i**, *i*-PrOH, HCl (cat.), microwave, 150 °C, 4 h (ii) 1 N NaOH, reflux, 4 h (*h*) (i) **10i**, *i*-PrOH, HCl (cat.), reflux, 6 days (ii) 1 N NaOH, reflux, overnight. (*i*) (i) **9i**, *i*-PrOH, HCl (cat.), microwave, 80 °C, 12 h (ii) 1 N NaOH, reflux, 4 h (*j*) (i) **5i**, *i*-PrOH, HCl (cat.), reflux, 72 h (ii) 1 N NaOH, reflux, 4 h (*k*) **4i**, *i*-PrOH, HCl (cat.), microwave, 80 °C, 7 h (ii) 1 N NaOH, reflux, 4 h.

Synthesis of compounds **1** and **2** has been described previously.^39^ Modifications to the reported procedure (Scheme 2), in this study, significantly enhanced the yields. For all steps this involved, variations of time and temperature and afforded yields that were increased by 5–10%. Compound **5a**^39^ was reduced with zinc dust to compound **6a**. Cyclo-condensation of compound **6a** with carbamimidic chloride hydrochloride afforded compound **7a**. The 2-amino substituent of **7a** was protected with 2,2-dimethyl propanoic anhydride to afford compound **8a**. Compound **9a** was synthesized from compound **8a** with phosphorus oxychloride at reflux. It was of interest to synthesize compound **9a** as displacement of the 4-Cl allows for the synthesis of varied analogs by utilizing amines obtained (compound **3ii**-**12ii**) *via* Scheme 1. Displacement of the 4-Cl of compound **9a** with appropriately substituted *N*-methyl aromatic amines (compound **1i**-**13i**) followed by base-mediated deprotection of the 2-NH_2_ group afforded the target compounds **1**–**13**. The yield of the final S_N_Ar displacement step was adversely influenced by the bulk of the aromatic amine, as expected. For example, bicyclic substitutions on the *N*-methyl amines (compounds **6i, 7i**, **11i** and **12i**) caused the S_N_Ar reaction to have a significantly reduced yield (18–30%) when compared (27%–69%) with monocyclic *N*-methyl substituted amines (compounds **1i-5i**, **8i-10i** and **13i**).

### Biological evaluation and discussion

Thus far, molecular modeling of compounds **1**–**13** suggested tubulin stabilization mediated by binding to the vinca binding site on tubulin, to produce a less curved (*i.e.*, stabilized) microtubule assembly. In addition, docked poses in pathologically implicated kinases suggested multiple targets, prompting further biological evaluation of compounds

### Compounds **1**–**13** exhibit binding to tubulin in vitro as well as inhibition of clinically relevant kinases in vitro

Table 1 shows the evaluations of the inhibition of appropriate kinases in isolated enzyme studies (ThermoFisher^51^). In addition, compounds **1**–**13** were evaluated for interactions with tubulin, as described.^52^ The molecular modeling predictions for the colchicine site and the vinca site on tubulin were found to be accurate for most of the compounds. Thus, except compounds **3** and **5**, the designed compounds display a lower binding to the colchicine site compared to compound **1.** This may be attributed to a better docked score that was observed for compounds **3** and **5** due to interactions with Cys241. Compounds **3, 5, 6** and **12** demonstrate an increased displacement of vinblastine (Table 1), which suggests better binding to the vinca site on tubulin than compounds **1** and **2**. The data in Table 1 also suggest that compounds **1** and **2** differ in MT dynamics, but both inhibit the kinases.

The unsubstituted phenyl of compound **1** is most active across the board. The *p*-tolyl substitution (**2**), and other bulky substituents (compounds **4**, **7–11** and **13**) caused a reduction in binding at both the colchicine and vinblastine site, which was not measurable when compared to compound **1**. Compounds **6** and **12** with fused bicyclic substitutions at the *N^4^*-position were detrimental to colchicine binding while displaying improved displacement of vinblastine at the vinca site (Table 1), as predicted from molecular modeling studies. The reduction in binding at the colchicine site by compounds **4** and **6–13** may be attributed to a snug binding pocket that is unable to accommodate substitutions on the *N^4^*-phenyl moiety. On the other hand molecular modeling indicates that, compounds **6** and **12** demonstrate an ≈ 8 Å shift in the α helix in the vinca binding site similar to compound **1;** however, compound **2** caused a shift of <7 Å while compounds **3–5**, **7–11** and **14** cause a shift of 7–8 Å. These modeling results corroborate the data obtained in Table 1 and indicate that bicyclic substitutions would be conducive to tubulin stability *via* vinca site binding.

Except for ABL1, compounds **1** and **10** were the best kinase inhibitor. Compound **6** with *N^4^*-bicyclic substitution would rank next, indicating that a restricted alkoxy function in the furan ring has reasonable relevant multi-kinase inhibitory properties when compared to the other compounds in Series I. Although, compound **2** offers weaker inhibition of the kinases, the poorest kinase inhibitors are compounds **8** and **11**, as predicted by the docked scores in molecular modeling (Table S1).

### Cytotoxicity of multi-kinase inhibitors and potential tubulin modulators compounds **1**–**13**

The data obtained using tumor cells in Table 2 indicates a lack of toxicity in cell culture for compounds **1**, **2**, **5**–**8** and **11**–**13**. This is significant because kinase inhibitors of LRRK2,^53^ ABL1,^54^ GSK3β,^55^ and DYRK1A^56^ have been developed as anticancer agents. Since the proposed compounds were designed to affect neuronal activity, it was imperative that these analogs were nontoxic to neuronal cells. However, the kinase inhibitory potency required for anticancer activity is *much higher* than that of compounds **1** and **2** (Table S2).

As our aim is mild inhibition of the relevant kinases along with subtle tubulin stabilization to avoid any neuronal cytotoxicity, it is important that agents such as compounds **1**, **2**, **5**–**8** and **11**–**13** *avoid* potent (<100 nM) kinase inhibition as well as potent tubulin stabilization, as these are attributes of cytotoxic antitumor agents. Though docked poses of compounds **1**–**13** did not vary by a significant extent, the cytotoxicity and tubulin modulation of all analogs varied significantly, which emphasizes that subtle structural manipulation can remarkably affect the biological activity of these compounds. While the molecular modeling successfully predicted the potential for activity across the series (consistent with compounds **1** and **2**), the *in vitro* activities of compounds **1**–**13** imply that factors outside the scope of the docking model, such as, the subtle balance between kinase and tubulin modulation, are critical for preventing cytotoxicity or inactivity.

On the basis of the kinase activity, the tubulin binding assays and the nontoxic nature of compounds **1** and **2** in tumor cell lines, we elected to evaluate them further in an established primary hippocampal culture model of LBD.^57–59^ Compounds **6** and **12** from Series I and II, respectively, were also chosen to be analyzed in this model based on the comparable kinase inhibition in their respective series and lack of cell toxicity. In this *in vitro* model, preformed fibrils are used to generate dense α-synucleinopathic inclusions within primary hippocampal neurons,^57–59^ as defined by hyperphosphorylated α-syn (pSer129 α-syn), the best available marker for Lewy bodies and Lewy neurites.^60,61^ Although the phosphorylation of α-syn at the serine 129 residue has physiological roles under baseline conditions (absence of pathology),^62,63^ there is a marked increase in this measure in LBD.^60,64–67^ We previously demonstrated that α-syn fibrils do not generate severe cell loss in mixed-sex postnatal hippocampal cultures within 10 to 14 days of exposure.^59^ The latter model was therefore employed to model *early-stage* α-synucleinopathy.

### Compounds **1** and **2** reduce α-synucleinopathy in primary hippocampal cultures

**Fig. 5.**
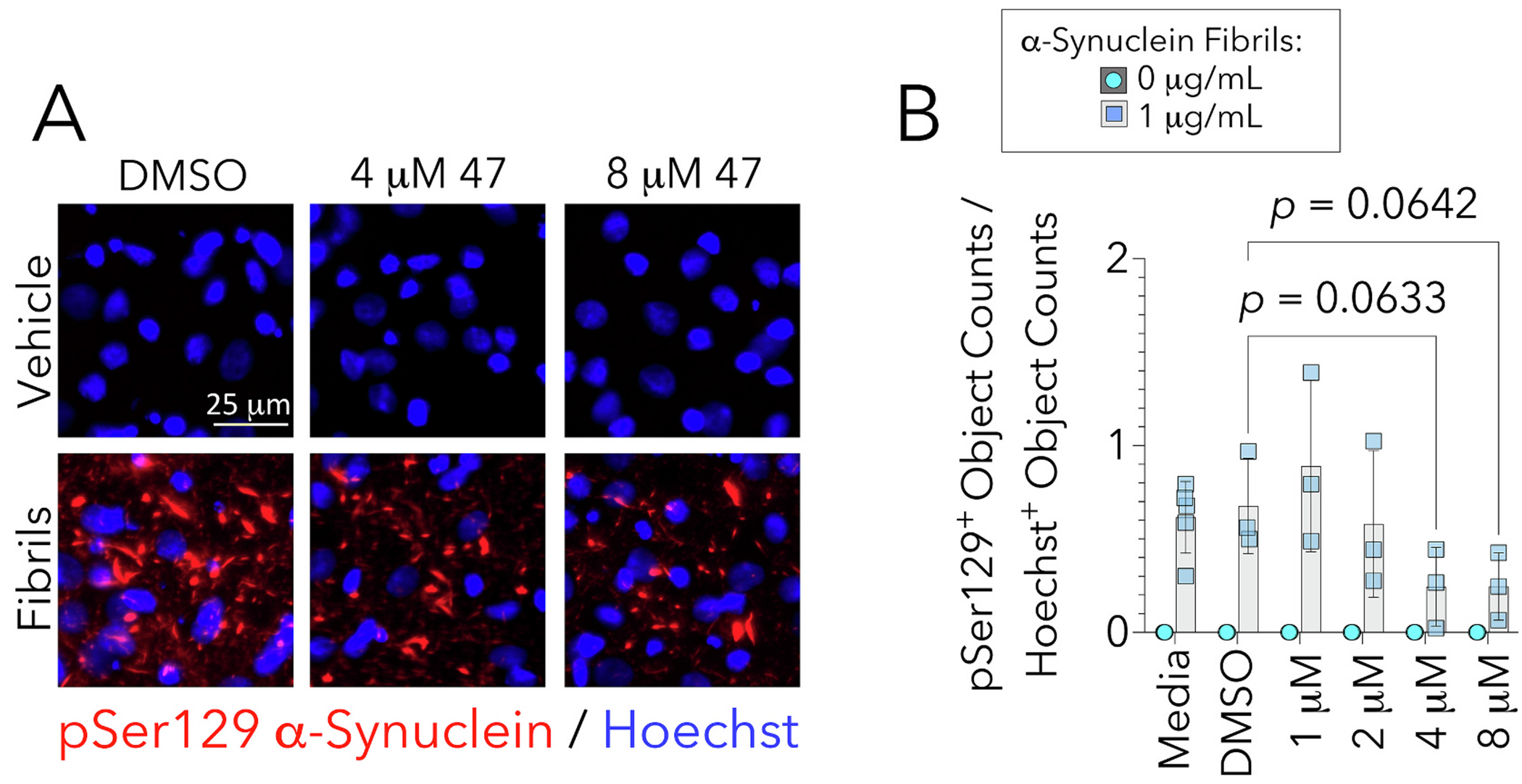
Impact of Compound **2** on α-synucleinopathy in primary hippocampal cultures. Primary hippocampal cultures were generated from postnatal rat pups and challenged with preformed α-syn fibrils at 1 μg/mL for 10 days *in vitro*. Cultures were simultaneously treated with escalating concentrations of the microtubule/kinase-targeting agent, Compound **1**, before fixation and immunostaining for hyperphosphorylated α-syn (pSer129), as an *in vitro* marker of Lewy-like pathology, and application of the pan-nuclear staining reagent Hoechst. (A) Representative images of pSer129 α-syn and Hoechst staining. Scale bar in A applies to all panels. (B) pSer129^+^ objects were expressed as a fraction of Hoechst^+^ nuclei and subjected to a repeated measures (*i.e.*, with matching for each independent culture) two-way ANOVA/Bonferroni analysis. Statistically significant effects of preformed fibrils *versus* vehicle-treated cultures were also observed at every concentration of Compound **1** (*p* ≤ 0.0500). Shown are the mean ± S.D. for *n* = 3 independent cultures, each run in duplicate or triplicate wells. Replicate wells from independent cultures were then averaged to yield a single number as the final statistical unit, plotted as a blue dot in the graph. (For interpretation of the references to colour in this figure legend, the reader is referred to the web version of this article.)

**Fig. 6.**
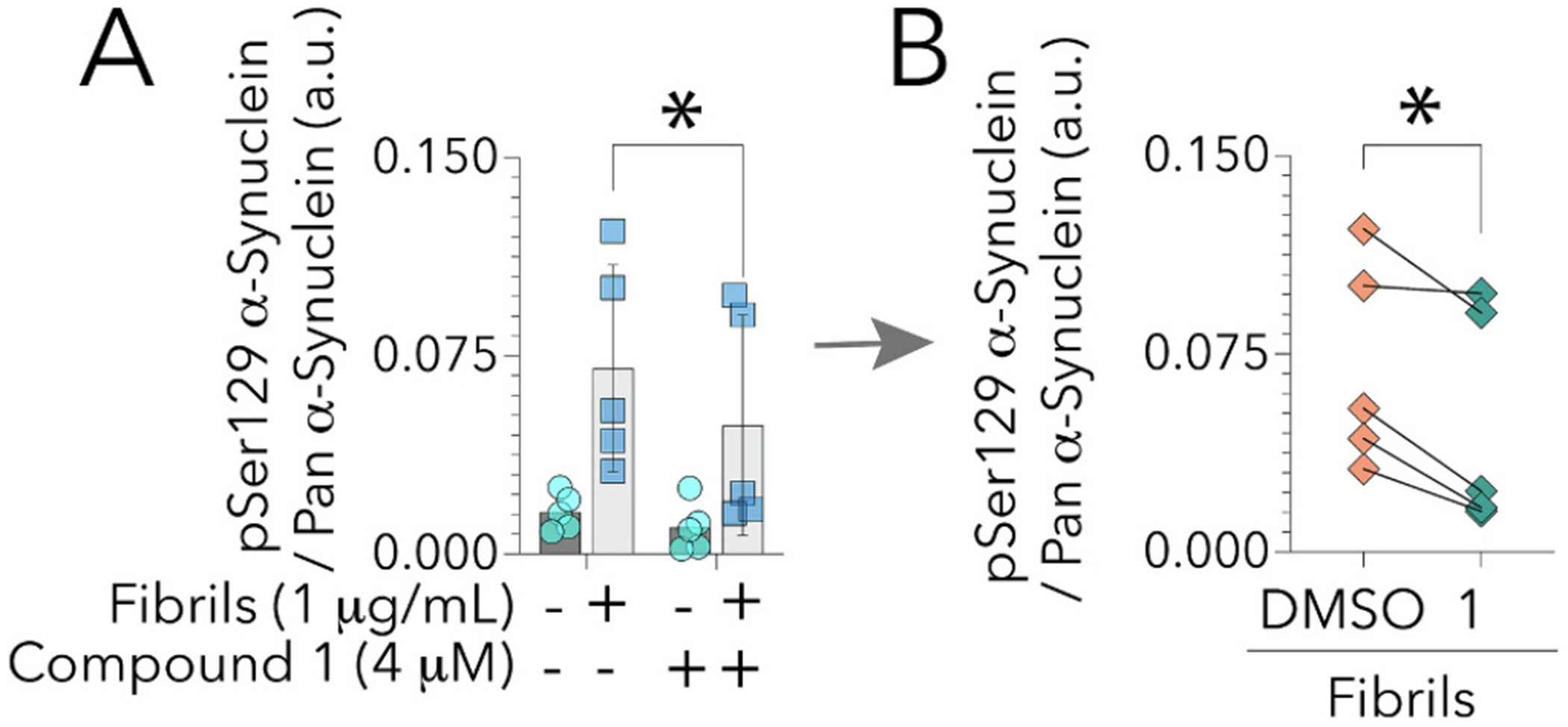
Impact of Compound **1** on hyperphosphorylation of α-synuclein. Primary rat hippocampal cultures were treated *in vitro* for 10 days with preformed fibrils and Compound **1** or respective vehicles. Levels of (A–B) pSer129 α-syn as a fraction of pan α-syn were probed by immunoblotting. Shown are the mean ± S.D. of *n* = 5 independent cultures for all groups, each run in a single well for immunoblotting (values shown as a dot in the graphs). *p ≤ 0.0500, repeated measures (*i.e.*, with matching for each independent culture) two-way ANOVA/Bonferroni in panel A, and a two-tailed paired *t*-test on the fibril-treated groups alone (follow arrows to panel B). Statistically significant effects of fibril exposure were also observed in A (*p* ≤ 0.0500). Full-length immunoblots are in Fig. S4. a.u. = arbitrary units.

We tested the influence of compound **1** on the pathological hyperphosphorylation of α-syn at the serine 129 residue (pSer129) in three types of assays (Figs. 5–6; Figs. S2–S4). In all three cases, a tendency towards a reduction in α-synucleinopathy was distinctively observed. This result preliminarily suggested that compound **1** has the potential to mitigate Lewy-related pathologies. In the immunoblotting analyses, levels of exogenous and endogenous pan α-syn levels were not affected by compound **1** (Fig. S4) but, importantly, the hyperphosphorylation of α-syn in the fibril-treated neurons was lowered at 4 μM by compound **1** (Fig. 6). As we have shown before,^59^ the α-synucleinopathic lesions stained by antibodies against pSer129 were resistant to the nonionic surfactant and emulsifier Triton X-100 (Fig. S2D).

**Fig. 7.**
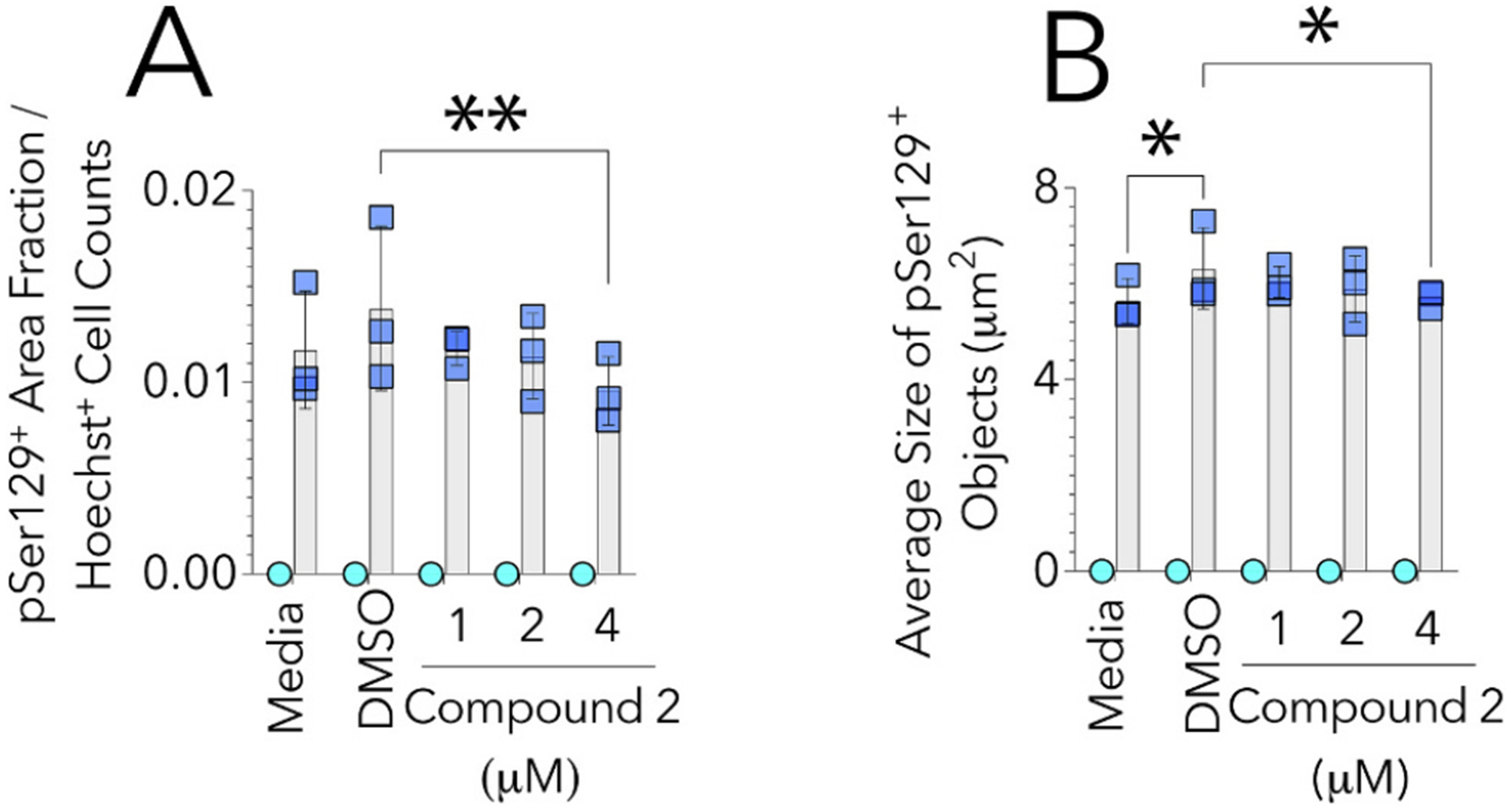
Impact of Compound **2** on α-synucleinopathy in primary hippocampal cultures. Primary rat hippocampal cultures were treated with **2** and preformed α-syn fibrils for 10 days *in vitro* before immunostaining for hyperphosphorylated α-syn (pSer129) and applying the pan-nuclear Hoechst reagent. (A) The area occupied by pSer129^+^ objects (area fraction) per Hoechst^+^ cell, and (B) average sizes of individual pSer129^+^ objects. Statistically significant effects of preformed fibrils *versus* vehicle-treated cultures were also observed at every concentration of Compound **2** (*p* ≤ 0.0500). Shown are the mean ± S.D. of n = 3 independent cultures, each run in duplicate or triplicate. Replicate wells from independent cultures were then averaged to yield a single number as the final statistical unit, plotted as a blue dot in the graphs. *p ≤ 0.0500, **p ≤ 0.0100, two-way repeated measures (with matching for each independent culture) ANOVA/Bonferroni. Representative images are in Fig. S2. (For interpretation of the references to colour in this figure legend, the reader is referred to the web version of this article.)

**Fig. 8.**
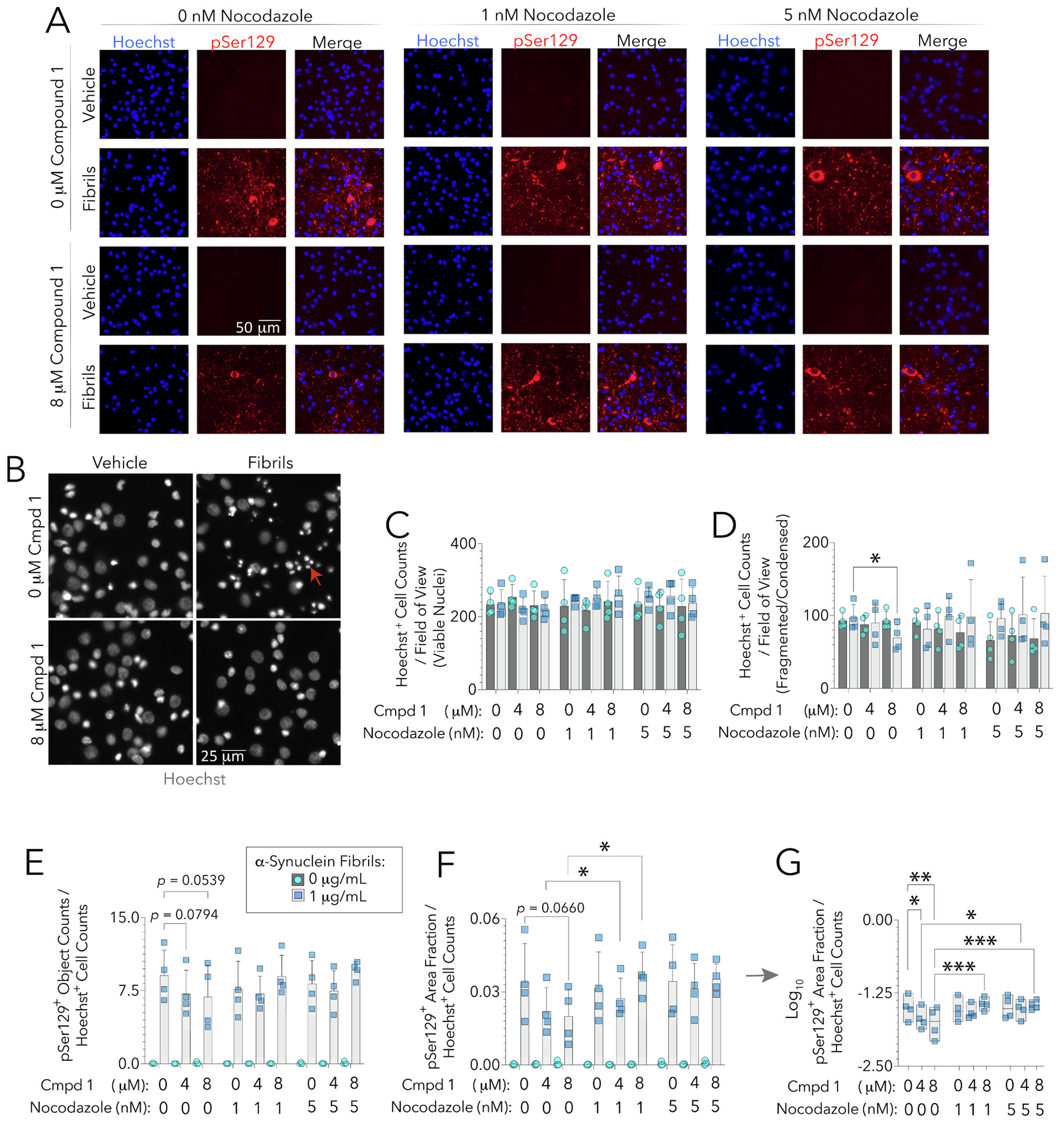
Impact of the microtubule destabilizer/depolymerizer nocodazole on the activities of Compound **1**. Primary rat hippocampal cultures were treated for 10 days *in vitro* with preformed fibrils, compound **1**, and nocodazole (or their respective vehicles). Representative images of hippocampal cultures (A) stained with the pan-nuclear marker Hoechst and immunolabeled for hyperphosphorylated α-syn (pSer129). (B) The red arrow marks examples of condensed/fragmented nuclei. Scale bars in subpanels of A and B apply to all images in panels A and B, respectively. (C) Viable Hoechst^+^ cell counts (see Supplementary Methods for morphometric definitions) per field of view under a 20× objective. (D) Nonviable Hoechst^+^ fragmented/condensed nuclei per field of view under a 20× objective. (E) pSer129^+^ object counts per Hoechst^+^ cell. (F) The area occupied by pSer129^+^ objects (area fraction) per Hoechst^+^ cell. (G) Log_10_ pSer129^+^ area fraction per Hoechst^+^ cell (the raw data are shown in panel F). Statistically significant effects of experimental Lewy body disease (*i.e.*, fibril exposure) were also observed in E–F (*p* ≤ 0.0500). Shown are the mean ± S.D. or boxplots for *n* = 4 independent cultures, each run in duplicate or triplicate wells. Replicate wells from independent cultures were averaged to yield a single number as the final statistical unit, plotted as a blue dot in the graphs. * *p* ≤ 0.0500, ** *p* ≤ 0.0100, *** *p* ≤ 0.0010, repeated measures (with matching for each independent culture) two-way ANOVA/Bonferroni. (For interpretation of the references to colour in this figure legend, the reader is referred to the web version of this article.)

We also tested compound **2** in primary hippocampal cultures. Here, the total area occupied by pSer129^+^ objects per cell and the average pSer129^+^ object size per cell were slightly reduced by exposure to compound **2** at 4 μM (Fig. 7A–B, S2). While compound **2** was poorly active in binding to the vinca binding site on tubulin, its kinase inhibitory activity was comparable to compound **1** and may be responsible for a reduction in α-synucleinopathy, albeit not as much as compound **1**, which binds to the vinca site and also inhibits pathologically relevant kinases. As is common for pharmacologic agents, high concentrations of the compounds were neurotoxic and, therefore, not used for calculations of pSer129^+^ load. It is also important to note that the mitigating impact on Lewy-related pathologies was observed at concentrations that did not elicit neurotoxicity, as is evident from all our calculations of Hoechst+ cell numbers as well as prevention of the fragmentation and atrophy of Hoechst+ nuclei in Fig. 8 (see below).

Compounds **6** and **12** were tested but exerted no consistent effects on neuronal viability or on pSer129^+^ α-synucleinopathy (Fig. S3). The inability of compound **6** to decrease pSer129^+^ objects was unanticipated from data in Table 1. While compound **6** was comparable to compound **1** as a multi-kinase inhibitor and bound to the vinca site to a greater extent than compound **1**, its inactivity in primary hippocampal cultures suggests that physicochemical properties such as cellular permeability and/or solubility may limit its intracellular efficacy. This would require further pharmacokinetic studies for confirmation. Similarly, while compound **12** demonstrated binding at the vinca site on tubulin, the lower potency of this compound in the pathologically relevant kinases (Table 1) may be responsible for the lack of consistent effects on pSer129^+^ α-synucleinopathy.

### The microtubule disruptor nocodazole impedes the activities of compound **1**

To ascertain if compound **1** impacted microtubules and expand our initial assessments of this agent, we tested if sublethal concentrations of the established microtubule destabilizer/depolymerizer nocodazole^68^ would reduce or abolish the activity of compound **1** on pSer129^+^ structures. Primary hippocampal cultures were treated with preformed fibrils and escalating concentrations of compound **1** and sublethal concentrations of nocodazole (1 and 5 nM; Fig. 8A–B). At the highest concentration (8 μM), compound **1** reduced fragmented and condensed Hoechst^+^ nuclei in the fibril-treated group (red arrows in Fig. 8B; quantified in Fig. 8D). This protective impact of compound **1** on fragmented/condensed cell counts in the fibril groups was prevented by both concentrations of nocodazole.

We also assessed whether the ability of compound **1** to reduce pSer129^+^ α-synucleinopathy in primary neurons would be prevented by nocodazole (Fig. 8A, EfragG). The number of pSer129^+^ object counts per cell was lowered by compound **1** in Fig. 8E. In addition, a log_10_-transformation of the non-Gaussian area fraction measurements revealed that nocodazole abolished the activity of compound **1** against pSer129+ α-synucleinopathic load (Fig. 8G). As nocodazole is an established microtubule disruptor,^68^ these observations support the idea that compound **1** reduces pSer129^+^ α-synucleinopathic load in part, *via* direct or indirect action on microtubule dynamics. It is also possible that the kinase inhibitory effects of compound **1** may play a role in the reduction of pSer129^+^ α-synucleinopathic load. Although the effects of compound **1** may be related to microtubule disruption, potential off-target effects of pharmacologic agents, such as nocodazole, cannot be ruled out.

### Impact of Compound **1** on microtubule stabilization markers

**Fig. 9.**
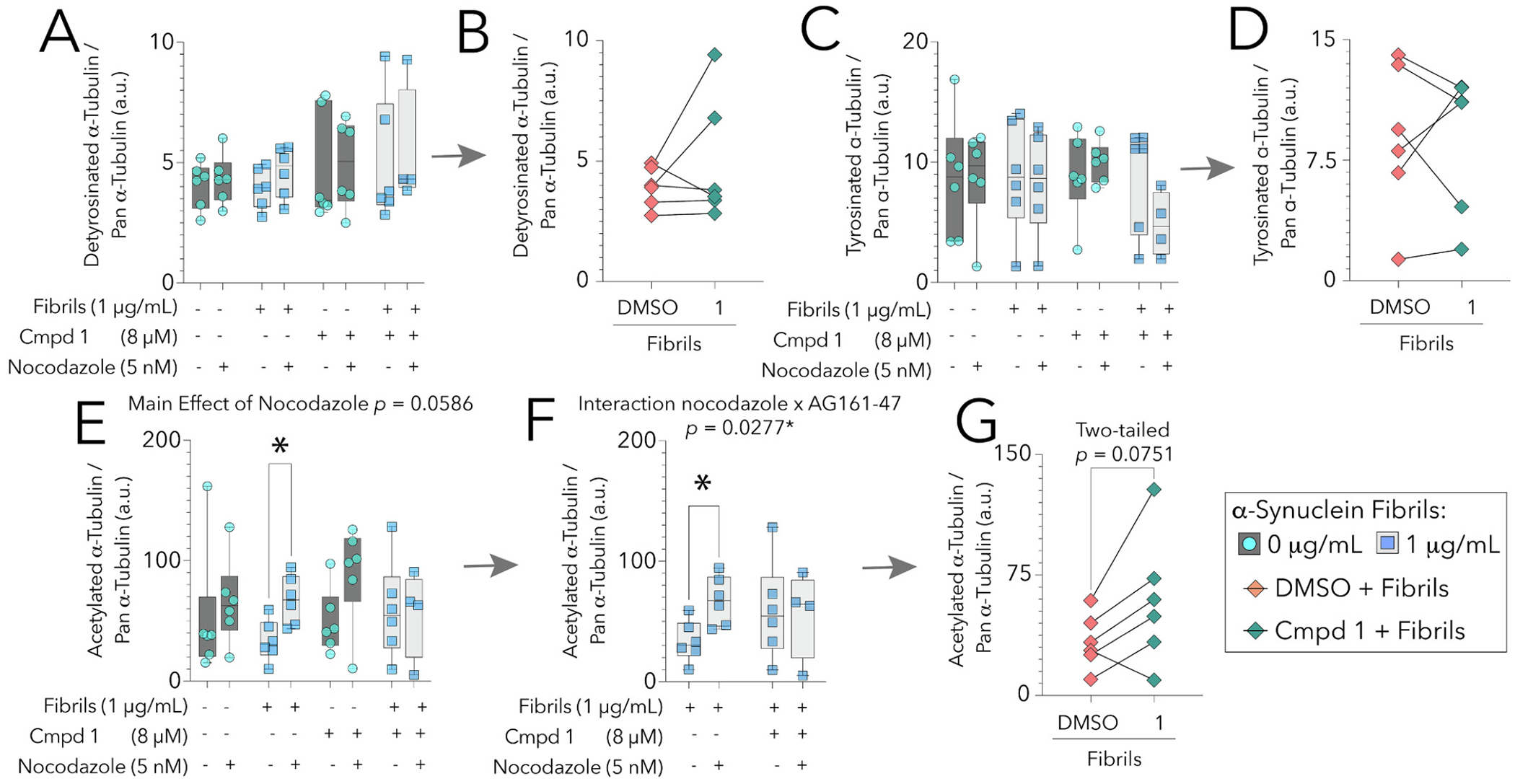
Impact of Compound **1** on post-translational markers of microtubule stability. Primary hippocampal cultures were treated for 10 days *in vitro* with preformed fibrils, Compound **1**, and nocodazole (or their respective vehicles). Quantification of the levels of (A–B) detyrosinated α-tubulin, (C–D) tyrosinated α-tubulin, and (E–F) acetylated α-tubulin as a fraction of pan α-tubulin. Shown are boxplots or scatterplots of 4–6 independent cultures, each run in a single well for immunoblotting and plotted as a dot in the graphs. * *p* ≤ 0.0500, repeated measures (with matching for each independent culture) two or three-way ANOVA/Bonferroni and two-tailed paired *t*-tests on fibril-treated groups alone (follow arrows). a.u. = arbitrary units. Full length blots are in Fig. S5.

To further evaluate how compound **1** affects microtubules, we used established markers of microtubule stabilization (detyrosination) and lack of stabilization (tyrosination).^69,70^ Detyrosination may not impart cytoskeletal stability *per se* but the probability of this post-translational modification rises in parallel with increased microtubule lifespan and is therefore naturally higher in the stable microtubule fraction.^69,71,72^ Conversely, non-detyrosinated (*i.e.*, tyrosinated) tubulin polymers are more commonly encountered within the non-stabilized fraction of neuronal microtubules.^71,72^ Compound **1** exerted no consistent effects on tyrosinated (nonstable) or detyrosinated (stable) α-tubulin by Western immunoblotting (Fig. 9A–D).

A post-translational modification that is more commonly observed in the stable fraction of microtubules is the acetylation of intraluminal lysine 40 (K40) of α-tubulin, catalyzed by α-tubulin *N*-acetyltransferase (ATAT1).^69,70^ Acetylation has been argued to impart microtubules with mechanical resistance, as ATAT1 depletion raises the probability of microtubule breakage.^73–75^ When fibril-treated groups were subjected to a focused analysis under conditions of experimental LBD (in the presence of fibrils but the absence of nocodazole) compound **1** showed a modest tendency to raise acetylated α-tubulin levels in fibril-treated groups (two-tailed *p* = 0.0751; Fig. 9G). The overall stabilizing effect was mild—which we view as a positive result. Robust microtubule stabilizer epothilone was severely toxic at nanomolar concentrations and, therefore, failed as a positive control in this *in vitro* model (data not shown). These findings on epothilone are consistent with prior work demonstrating that excessive rigidity of microtubules prohibits microtubule dynamicity and exerts toxicity.^76,77^

### Compounds **1**, **2**, **6** and **12** demonstrate a favorable pharmacokinetic (PK) profile for brain penetrability

Compounds **1**, **2**, **6** and **12** must traverse the blood-brain barrier (BBB). An *in-silico* indication (Qikprop) of brain penetrability is defined by log BB, a ratio of the drug concentration in brain *vs* plasma. Schrödinger^44^ defines a log BB in the range of −3.0 to 1.2 as necessary to penetrate the BBB with a more positive value suggesting higher permeability. We verified this range for compounds **1** and **2** in preclinical animal models. Recent *in vivo* results of BBB penetration (Table 3) for compounds **1** and **2** (brain/plasma ratio (Kp brain) of 4.50 and 5.99 respectively) in CD-1 mice validates Schrödinger predictions of compounds **1** and **2**, providing credence to the predicted BBB penetration for compounds **6** and **12**. In Table 3, log BB values for compounds **1**, **2** (verified *in vivo*), and compounds **6** and **12** are all within range.

Based on the data obtained, we elected to evaluate compound **1** in preliminary animal studies. As expected, the pilot studies indicate a modest decrease in hyperphosphorylated, Triton-insoluble α-syn in the CNS after P.O. administration of 5 mg/kg compound **1**, in mice infused with preformed fibrils in the olfactory bulb/anterior olfactory nucleus.

## Conclusion

In summary, molecular modeling and docking scores for tricyclic analogs **1**–**13** supported further evaluation of these multitargeted but *appropriately* mild inhibitors of i) vinblastine binding for dynamic tubulin stabilization and ii) kinases critically involved in hyperphosphorylation of tau and α-syn. It is important to note that both activities—stabilization of tubulin *and* inhibition of pertinent kinases—must be mild. Potent effects at either site could result in undesired neurotoxicity. The biological evaluation suggested that compound **1** binds at the tubulin vinca site and nudges microtubule dynamics without overstabilizing tubulin, thereby avoiding the frank neurotoxicity we observed with epothilone. In addition, compound **1** decreased the levels of hyperphosphorylated α-syn *in vitro* and *in vivo*. In primary hippocampal cultures treated with preformed fibrils, the moderating impact of compound **1** was prevented by the microtubule disruptor nocodazole. Further animal studies are in progress to comprehensively evaluate the mechanism of action and *in vivo* effects of these microtubule/kinase modulators. On the basis of our results, we propose compounds **1** and **2** as the first-in-class multitargeted tubulin modulators and kinases inhibitors—as new leads for further optimization in the treatment of neurodegenerative disorders.

## Supplementary Material

Supplemental Materials

## Figures and Tables

**Table 1:** *In vitro* inhibition data for compounds **1–4** for tubulin and pertinent kinases.

Compound	% Inhibition of colchicine binding at 100 μM inhibitor	% Inhibition of vinblastine binding at 50 μM inhibitor	Average % inhibition of kinase at 10 μM inhibitor			
			ABL1	DYRK1A	GSK3β	LRRK2
**1**	34	15	56.5	48	61	59
**2**	Very poorly active*	Very poorly active*	42	21	27	49
**3**	86	15	31	27	21	36
**4**	Very poorly active*	Very poorly active*	50	10	19	31
**5**	52	17	30	13	26	29
**6**	28	24	67.5	41	40.5	47
**7**	Very poorly active*	Very poorly active*	47	13	25	12
**8**	Very poorly active*	ND	18	13	19	16
**9**	Very poorly active*	ND	27	17	25	29
**10**	Very poorly active*	ND	66.5	24	38.5	64.5
**11**	ND	ND	8	8	12	9
**12**	19	18	20	14	21	34
**13**	Very poorly active*	ND	47	13	15	12

An asterisk* signifies that the signal to noise ratio is below detection threshold.

ND = not determined.

**Table 2:** *In vitro* cytotoxicity data for compounds **1–13**.

Compound	Inhibition of cell growth IC_50_ ± SD (nM)		
	OVCAR 8	NCI/ADR-RES	MCF-7
**1**	3900 ± 900	4400 ± 900	4200 ± 1000
**2**	>5000	>5000	>5000
**3**	220 ± 50	130 ± 30	330 ± 60
**4**	170 ± 20	80 ± 3	170 ± 30
**5**	>5000	>5000	>5000
**6**	3200 ± 1000	4300 ± 500	2900 ± 200
**7**	>5000	>5000	>5000
**8**	>5000	>5000	>5000
**9**	140 ± 10	82 ± 9	110 ± 30
**10**	290 ± 10	360 ± 30	320 ± 4
**11**	>5000	>5000	>5000
**12**	>5000	>5000	>5000
**13**	>5000	>5000	>5000

**Table 3:** *In vivo* PK profile of compounds **1** and **2** and predicted PK property for com pounds **6** and **12**.

Compound	Schrödinger predicted log BB value	*In vivo PO* PK study in CD-1 mice				
		Time (h)	Brain (ng/mL)	Plasma(ng/mL)	Brain/Plasma ratio (Kp brain)	Mean
**1**	−0.737	2	48.4	8.93	5.4	4.5
			46.0	12.9	3.5	
			47.2	10.4	4.5	
**2**	−0.776	2	139	23.0	6.05	5.99
			158	26.7	5.93	
			137	22.6	6.07	
**6**	−0.717	Not determined				
**12**	−0.679					

## Data Availability

Data will be made available on request.

## Appendix A. Supplementary data

Supplementary data to this article can be found online at https://doi.org/10.1016/j.bmcl.2026.130536.
