# Supplementary material for: Discovery of multitargeting single agents as a novel route to the potential treatment of neurodegenerative diseases
Source: Bioorg Med Chem Lett. Author manuscript; Available in PMC 2026 Jun 13. (PMC13263999; doi:10.1016/j.bmcl.2026.130536)
Supplement: Supplemental Materials [file NIHMS2180182-supplement-Supplemental_Materials.docx]

**Supplementary information**

**Discovery of multitargeting single agents as a novel route to the potential treatment of neurodegenerative diseases**

Jeetal Vyas^1#^, Anuj S. Jamenis^1#^, Krishna Kaku^1^, Yesha Shah^1^, Kristin M. Miner^1^, Tarun N Bhatia^1^, Roxanne E. Kim^1^, Ruoli Bai^2£^, Ernest Hamel^2^, Rehana K. Leak^1*^, Aleem Gangjee^1*^

^1^Division of Pharmaceutical Sciences, Duquesne University, Pittsburgh, PA

^2^Molecular Pharmacology Branch, Developmental Therapeutics Program, Division of Cancer Treatment and Diagnosis, Frederick National Laboratory for Cancer Research, National Cancer Institute, National Institutes of Health, Frederick, Maryland 21702, United States

*Corresponding Authors:

1. Aleem Gangjee, Ph.D.

451 Mellon Hall

Discipline of Medicinal Chemistry

School of Pharmacy

Duquesne University

Pittsburgh, PA 15219

Phone: 412.396.6070

[gangjee@duq.edu](mailto:gangjee@duq.edu)

1. Rehana K. Leak, Ph.D.

407 Mellon Hall

Discipline of Pharmacology

School of Pharmacy

Duquesne University

Pittsburgh, PA 15219

Phone: 412.396.4734

[leakr@duq.edu](mailto:leakr@duq.edu)

Notes: ^£^R.B.: Deceased June 25, 2024.

^#^J.V. and A.S.J.: contributed equally to the manuscript

Table of contents

1. Molecular modelling of **2**, **6** and **12** in various targets
2. Docked scores of compounds **1**,2 6 and 12 in various targets
3. Kinase inhibition data for **1** and **2**
4. Synthetic procedures
5. Experimental procedures and pharmacological methods
6. *
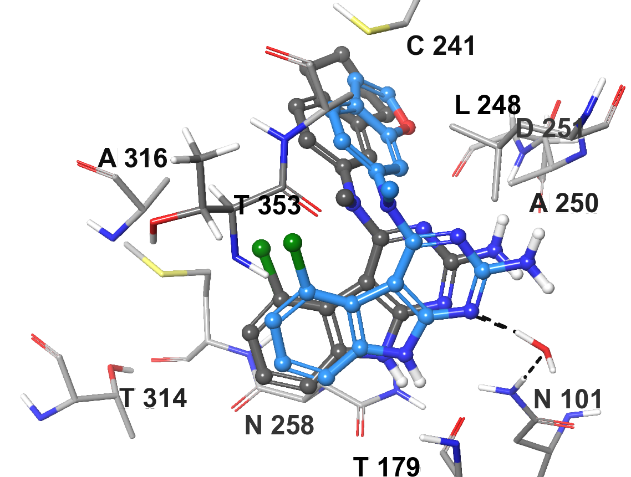
Molecular modelling of* ***6*** *and* ***12*** *in various targets*

[B]

[A]


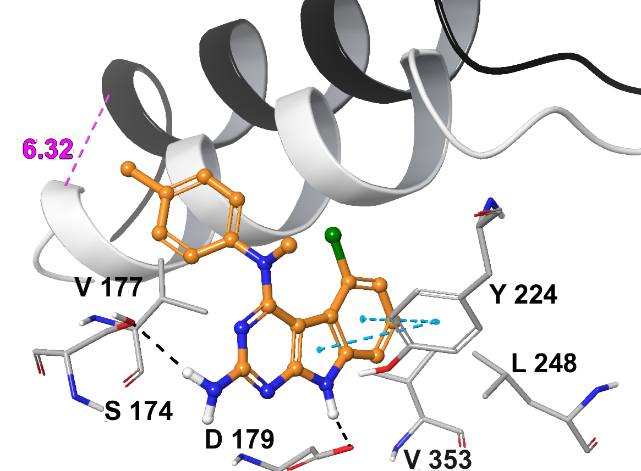


**6.32 Å**

*
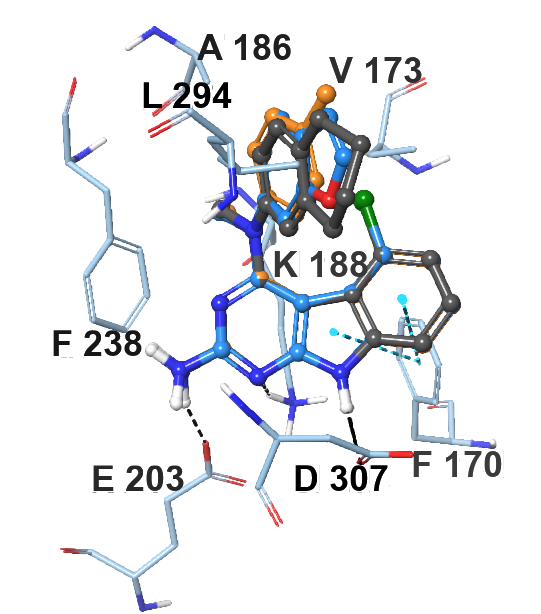

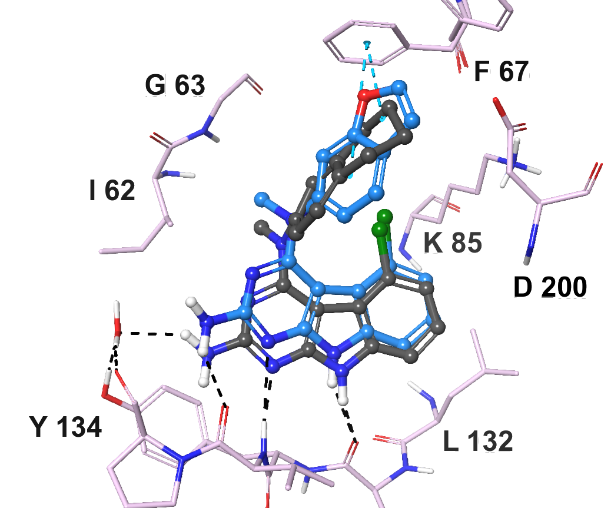
*

[D]

[C]

[F]

[E]

*
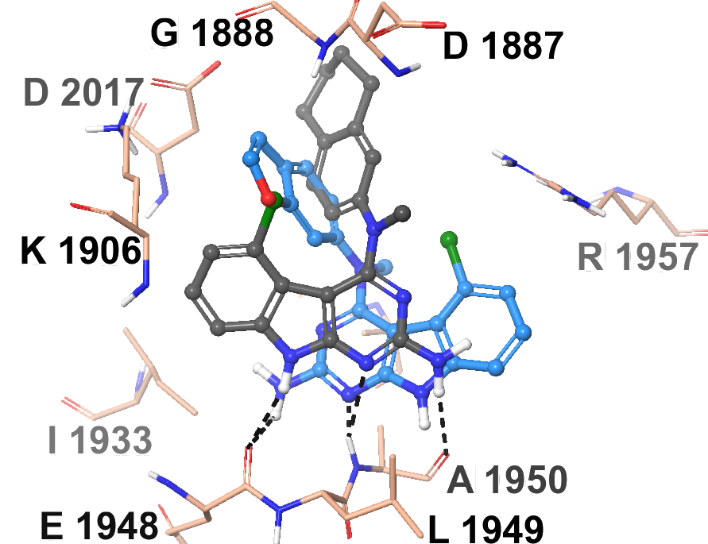
*
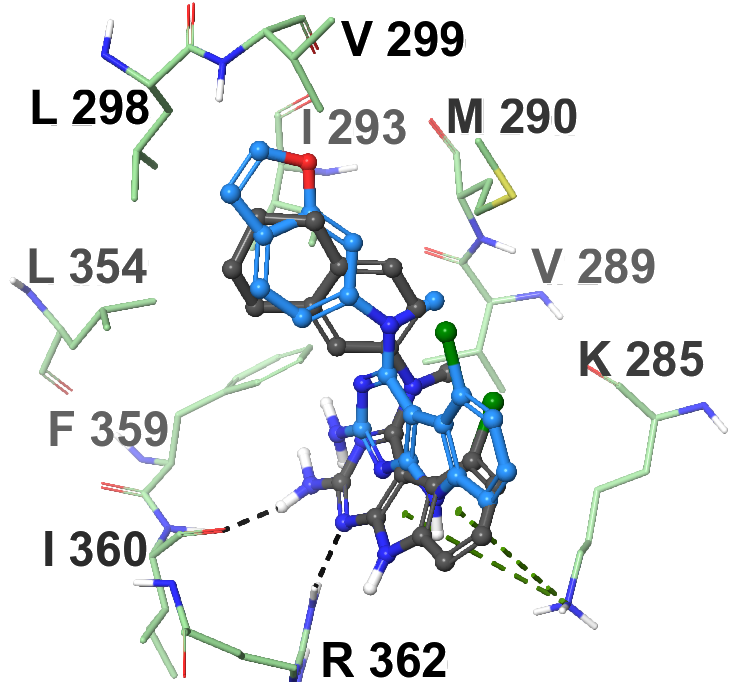


**Figure S1**. Docked poses of compounds **2**, **6** and **12** in different targets. (A) Docked pose of **6** (azure) and **12** (gray) in Colchicine binding site of tubulin (PDB ID: 4O2B). (B) Docked pose of **2** (faded orange) in Vinca binding site of tubulin (PDB ID: 5NJH, gray) superimposed with crystal structure of vinblastine (not shown) bound tubulin (PDB: 5J2T, black). (C) Docked pose of **6** (azure) and **12** (gray) in GSK3β (PDB ID: 7B6F). (D) Docked pose of compound **2** (faded orange), **6** (azure) and **12** (gray) in the binding site of DYRK1A (PDB ID: 7OY6) (E) Docked pose of compound **6** (azure) and **12** (gray) in ABL1 (PDB ID: 3CS9). (F) Docked pose of compound **6** (azure) and **12** (gray) in LRRK2 binding site (PDB ID: 8U7H).

1. *Docked scores of compounds* ***1****,* ***2****,* ***6*** *and* ***12*** *in various targets*

| Compound | Docked scores (kcal/mol) | | | | | |
| --- | --- | --- | --- | --- | --- | --- |
|  | Colchicine (PDB ID: 4O2B) | Vinca (PDB ID: 5NJH) | GSK3β (PDB ID: 7B6F) | ABL1 (PDB ID: 3CS9) | DYRK1A (PDB ID: 7OY6) | LRRK2 (PDB ID: 8U7H) |
| **1** | -10.891 | -9.324 | -10.865 | -7.365 | 9.287 | -9.785 |
| **2** | -9.994 | -9.444 | -8.903 | -6.599 | -7.435 | -9.649 |
| **3** | -9.556 | -10.869 | -8.992 | -6.344 | -7.856 | -9.112 |
| **4** | -9.645 | -10.992 | -8.882 | -7.665 | -7.655 | -9.342 |
| **5** | -8.355 | -9.666 | -9.001 | -6.662 | -7.009 | -8.992 |
| **6** | -9.438 | -10.236 | -10.021 | -7.549 | -8.606 | -10.011 |
| **7** | -8.992 | -9.891 | -8.991 | -6.745 | -7.665 | -8.431 |
| **8** | -8.765 | -9.543 | -10.943 | -6.445 | -7.232 | -8.776 |
| **9** | -8.254 | -9.553 | -9.872 | -6.893 | -7.453 | -9.954 |
| **10** | -8.365 | -9.756 | -10.002 | -7.774 | -6.912 | -9.996 |
| **11** | -9.001 | -9.452 | -9.034 | -5.453 | -7.009 | -8.001 |
| **12** | -9.364 | -10.133 | -9.113 | -6.444 | -7.463 | -7.226 |
| **13** | -9.132 | -9.889 | -9.025 | -6.981 | -6.491 | -7.923 |

**Table S1**. Docked scores of **1**, **2**, **6** and **12** in tubulin and pertinent kinases

| **Kinase**​ | **IC50 (µM) at Km app ATP**​ | |
| --- | --- | --- |
|  | **1** | **2** |
| ABL1​ | 7.96​ | 26.1​ |
| EGFR (ErbB1)​ | >10 | -​ |
| FGFR2​ | >10 | -​ |
| GSK3B (GSK3beta)​ | 8.12​ | 3.12​ |
| KDR (VEGFR2)​ | >10 | -​ |
| PDGFR (PDGFRbeta)​ | >10 | -​ |
| LRRK2​ | 7.03​ | 20.0​ |
| CSNK1A1 (CK1 alpha 1)​ | 30.5​ | 498​ |
| CSNK2A2 (CK2 alpha 2)​ | >1110​ | >1110​ |
| DYRK1A​ | 8.15​ | 249​ |
| FYN​ | 888​ | 169​ |
| GRK5​ | >1110​ | >370​ |
| GRK6​ | 614​ | 64.3​ |
| PLK2​ | >370​ | 16.7​ |

1. *Kinase inhibition data for* ***1*** *and* ***2***

**Table S2.** Kinase inhibition data for **1** and **2**

1. *Primary Antibodies:*

| **Antigen** | **Host** | **Manufacturer** | **Cat. No.** | **Lot. No.** | **Dilution** |
| --- | --- | --- | --- | --- | --- |
| Pan ⍺-tubulin | Rabbit | Sigma (Clone RM113) | SAB5600206 | S0602286, V0405082 | 1:1000 |
| Pan ⍺-tubulin | Mouse | Sigma (Clone B-5-1-2) | T5168 | 84283 | 1:100,000 |
| Tyrosinated ⍺-tubulin | Mouse | Sigma (Clone TUB-1A2) | T9028 | 029M4897V | 1:800 |
| Detyrosinated ⍺-tubulin | Rabbit | Millipore | AB3201 | 3584332, 3536025 | 1:500 |
| Acetylated ⍺-tubulin | Mouse | Sigma (Clone 6-11B-1) | T7451 | 078M4842V | 1:10,000 |
| pSer129 ⍺-synuclein | Rabbit | Abcam (Clone EP1536Y) | AB51253 | GR3232346-14 GR3317474-4 GR3191908-7 | 1:1000 |
| Pan ⍺-Synuclein | Mouse | BD Biosciences (Clone 42) | AB610786 | 0314065 | 1:1000 |
| β-III tubulin | Rabbit | Cell Signaling Technologies  (Clone D71G9) | 5568S | 6 | 1:300 |
| β-III tubulin | Chicken | Aves Labs | TUJ | TUJ89947984 | 1:2000 |
| MAP2 | Mouse | Sigma (Clone HM-2) | M9942 | 018M4785V | 1:750 |

**Table S3**. *Primary antibodies*

1. *Secondary Antibodies:*

| Antigen | Tag | Host | Manufacturer | Cat. No. | Lot. No. | Dilution |
| --- | --- | --- | --- | --- | --- | --- |
| Rabbit IgG | Alexa Fluor 790 | Donkey | Jackson ImmunoResearch Laboratories Inc. | 711-655-152 | 132235 | (WB) 1:15,000-30,000  (ICC) 1:700 |
| Rabbit IgG | Alexa Fluor 680 | Donkey | Jackson ImmunoResearch Laboratories Inc. | 711-625-152 | 135980 | (WB) 1:15,000-30,000  (ICC) 1:700 |
| Rabbit IgG | Cy3 | Donkey | Jackson ImmunoResearch Laboratories Inc. | 711-165-152 | 154879 | (WB) 1:15,000-30,000  (ICC) 1:700 |
| Mouse IgG | Alexa Fluor 790 | Donkey | Jackson ImmunoResearch Laboratories Inc. | 715-655-151 | 133569 | (WB) 1:15,000-30,000  (ICC) 1:700 |
| Mouse IgG | Alexa Fluor 680 | Donkey | Jackson ImmunoResearch Laboratories Inc. | 715-625-151 | 127891 | (WB) 1:15,000-30,000  (ICC) 1:700 |
| Mouse IgG | Alexa Fluor 488 | Donkey | Jackson ImmunoResearch Laboratories Inc. | 715-545-150 | 130996 | (WB) 1:15,000-30,000  (ICC) 1:700 |
| Chicken IgY | Alexa Fluor 647 | Donkey | Jackson ImmunoResearch Laboratories Inc. | 703-605-155 | 138591 | (WB) 1:15,000-30,000  (ICC) 1:700 |
| Chicken IgY | Alexa Fluor 680 | Donkey | Jackson ImmunoResearch Laboratories Inc. | 703-625-155 | 136950 | (WB) 1:15,000-30,000  (ICC) 1:700 |
| Rabbit IgG | Horseradish Peroxidase | Donkey | Jackson ImmunoResearch Laboratories Inc. | 711-035-152 | 157399 | (IHC) 1:160 |

**Table S4**. *Secondary Antibodies*

1. *Synthetic procedures:*

Chemicals were purchased at the highest purity available from ThermoFisher Scientific or Sigma-Aldrich. All evaporations were carried out in vacuum with a rotary evaporator. Analytical samples were dried *in vacuo* in a CHEM-DRY drying apparatus over P_2_O_5_ at 50 ^°^C. Melting points were determined either using a MPA100 OptiMelt automated melting point system and are uncorrected. Nuclear magnetic resonance spectra for proton (^1^H NMR) were recorded on the Bruker Avance II 400 (400 MHz) or Bruker Advance II 500 (500 MHz) NMR systems with TopSpin processing software. ^1^H spectra were referenced to tetramethylsilane and trifluoroacetic acid, respectively, as the internal standards to express the chemical shift values (*δ*) in ppm (parts per million): s, singlet; d, doublet; dd, doublet of doublet; t, triplet; q, quartet; m, multiplet; br, broad singlet; td, triplet of doublet; dt, doublet of triplet; quin, quintet. ^1^H NMR of alkyl CH_2_s of target compounds merged with dimethyl sulfoxide (DMSO) and water peaks have been omitted from the experiment whenever not visible in ^1^H NMR.

Thin-layer chromatography (TLC) was performed on Whatman® PE SIL G/UV254 flexible silica gel plates and the spots were visualized under 254 and 365 nm ultraviolet illumination. Proportions of solvents used for TLC are by volume. All analytical samples were homogeneous on TLC in at least two different solvent systems. Column chromatography was performed on the silica gel (70 to 230 meshes, ThermoFisher Scientific) column. Flash chromatography was carried out on the CombiFlash® *Rf* systems, model COMBIFLASH *RF*. Pre-packed RediSep® *Rf* normal-phase flash columns (230 to 400 meshes) of various sizes were used. The amount (weight) of silica gel/celite for column chromatography was in the range of 5-10 times the amount (weight) of the crude reaction mixture being separated. Mass spectrometry was performed on Advion expression® compact mass spectrometers (CMS). High Performance Liquid Chromatography (HPLC) was performed on Vanquish Core HPLC System (ThermoFisher Scientific) components: Multiwavelength detector CG (model code: VC-D12-⍺-01), Split sampler C (model code: VC-A13-A-02), Quaternary pump C (model code: VC-P20-A-01), Column compartment C (model code: VC-C10-A-03), Fraction Collector F (model code: VF-F11-A-01). The mobile phase was an aqueous blend of 0.1 % formic acid in water with a miscible, polar organic solvent acetonitrile.

**4-ethoxy-*N*-methylaniline (3i)**

4-ethoxyaniline (500 mg, 3.64 mmol) was added to a solution of NaOMe (984 mg, 18.22 mmol) in 10 mL MeOH. The resulting solution was poured into a suspension of paraformaldehyde (153 mg, 5.10 mmol) in MeOH (10 mL). The reaction mixture was stirred overnight at room temperature. Sodium borohydride (207 mg, 5.47 mmol) was then added, and the reaction mixture was heated at reflux for 5 hours. After cooling, silica gel was added to the reaction mixture and the solvent was removed under reduced pressure to provide a plug. The plug was transferred on top of a column packed with silica gel and was eluted with hexane:ethyl acetate as the eluent. Fractions containing the product (TLC) were pooled and evaporated to afford 226 mg (41 %) of **3i** as a dark green liquid. TLC *R_f_* = 0.63 (EtOAc:hexane 1:2 and 2 drops NH_4_OH); ^1^H NMR (500 MHz, DMSO-*d*_6_) δ 6.75 – 6.69 (m, 2H), 6.50 – 6.44 (m, 2H), 5.17 (s, 1H), 3.90 – 3.85 (m, 2H), 2.62 (s, 3H), 1.27 (t, *J* = 6.9 Hz, 3H).

**4-methoxy-*N*,3-dimethylaniline (4i)**

4-methoxy-3-methylaniline (1.37 g, 10 mmol) was added to a solution of NaOMe (2.7 g, 50 mmol) in 20 mL of MeOH. The resulting solution was poured into a suspension of paraformaldehyde (420 mg, 14 mmol) in MeOH (10 mL). The reaction mixture was stirred overnight at room temperature. Sodium borohydride (378.3 mg, 10 mmol) was then added, and the reaction mixture was heated at reflux for 2 hours. After completion of the reaction, 1M KOH was added to the reaction mixture and the product was extracted using EtOAc. The combined organic layers were dried over sodium sulfate and silica gel was added. Next, the solvent was removed under reduced pressure to provide a plug. The plug was transferred on top of a column packed with silica gel and was eluted with hexane:ethyl acetate. Fractions containing the product (TLC) were pooled and evaporated to afford 1.11 g (74 %) of **9i** as a red liquid. TLC *Rf* = 0.5 (EtOAc:hexane, 1:2); ^1^H NMR (400 MHz, DMSO-*d*_6_) δ 6.71 (d, *J* = 8.6 Hz, 1H), 6.39 (dd, *J* = 2.8, 0.8 Hz, 1H), 6.32 (dd, *J* = 8.6, 2.9 Hz, 1H), 5.12 (s, 1H), 3.66 (s, 3H), 2.61 (s, 3H), 2.07 (s, 3H).

***N*-methyl-4-(methylthio)aniline (5i)**

4-(methylthio)aniline (0.31 ml, 2.5 mmol) and NaH (90 mg, 3.75 mmol) were dissolved in THF at 0-5 °C. To this solution, MeI (0.23 ml, 3,75 mmol) was added and the resulting mixture was stirred at °C for an additional 3 hours. The reaction mixture was carefully quenched with water and was diluted with EtOAc. Organic layer was separated and washed with water (2-10 ml). The combined organic layers were dried over sodium sulfate and silica gel was added. Next, the solvent was removed under reduced pressure to provide a plug. The plug was transferred on top of a column packed with silica gel and was eluted with hexane:ethyl acetate. Fractions containing the product (TLC) were pooled and evaporated to afford 300 mg (78 %) of **5i** as a black liquid. TLC *Rf* = 0.5 (EtOAc:hexane, 1:4); ^1^H NMR (400 MHz, DMSO-*d*_6_) δ 7.15 – 7.10 (m, 2H), 6.53 – 6.48 (m, 2H), 5.72 (s, 1H), 2.67 – 2.63 (m, 3H), 2.34 (d, *J* = 0.4 Hz, 3H).

***N*-methylbenzofuran-5-amine (6i)**

Benzofuran-5-amine (500 mg, 3.76 mmol; CC07114DA, ThermoFisher Scientific) was added to a solution of NaOMe (1.1 g, 18.78 mmol) in 10 mL MeOH. The resulting solution was poured into a suspension of paraformaldehyde (157.85 mg, 5.26 mmol) in MeOH (10 mL). The reaction mixture was stirred overnight at room temperature. Sodium borohydride (142 mg, 3.76 mmol) was then added, and the reaction mixture was heated at reflux for 5 hours. After cooling, silica gel was added to the reaction mixture and the solvent was removed under reduced pressure to provide a plug. The plug was transferred on top of a column packed with silica gel and was eluted with hexane:ethyl acetate as the eluent. Fractions containing the product (TLC) were pooled and evaporated to afford 356 mg (64 %) of **6i** as a brown liquid. TLC *Rf* = 0.32 (EtOAc:hexane 1:4 and 2 drops NH_4_OH); ^1^H NMR (500 MHz, DMSO-*d*_6_) δ 7.79 (d, *J* = 2.1 Hz, 1H), 7.29 (d, *J* = 8.7 Hz, 1H), 6.75 (dd, *J* = 2.1, 1.0 Hz, 1H), 6.65 – 6.58 (m, 2H), 5.46 (q, *J* = 5.2 Hz, 1H, exch), 2.68 (d, *J* = 5.2 Hz, 3H).

***N*-methylbenzo[*b*]thiophen-5-amine (7i)**

Benzo[*b*]thiophen-5-amine (500 mg, 3.35 mmol) was added to a solution of NaOMe (905 mg, 16.75 mmol) in 10 mL MeOH. The resulting solution was poured into a suspension of paraformaldehyde (141 mg, 4.69 mmol) in MeOH (10 mL). The reaction mixture was stirred overnight at room temperature. Sodium borohydride (127 mg, 3.35 mmol) was then added, and the reaction mixture was heated at reflux for 5 hours. After cooling, silica gel was added to the reaction mixture and the solvent was removed under reduced pressure to provide a plug. The plug was transferred on top of a column packed with silica gel and was eluted with hexane:ethyl acetate as the eluent. Fractions containing the product (TLC) were pooled and evaporated to afford 150 mg (28 %) of **7i** as a dark red liquid. TLC *R_f_* = 0.47 (EtOAc:hexane 1:2 and 2 drops NH_4_OH); ^1^H NMR (400 MHz, Chloroform-*d*) δ 7.65 (dd, *J* = 8.6, 0.7 Hz, 1H), 7.40 (d, *J* = 5.4 Hz, 1H), 7.22 (dd, *J* = 5.4, 0.8 Hz, 1H), 7.01 (d, *J* = 2.3 Hz, 1H), 6.77 (dd, *J* = 8.6, 2.3 Hz, 1H), 2.92 (s, 3H).

***N*,3-dimethyl-4-nitroaniline (8i)**

3-methyl-4-nitroaniline (500 mg, 3.29 mmol) was added to a solution of NaOMe (887 mg, 16.43 mmol) in 10 mL MeOH. The resulting solution was poured into a suspension of paraformaldehyde (138 mg, 4.60 mmol) in MeOH (10 mL). The reaction mixture was stirred overnight at room temperature. Sodium borohydride (124 mg, 3.29 mmol) was then added, and the reaction mixture was heated at reflux for 5 hours. After cooling, silica gel was added to the reaction mixture and the solvent was removed under reduced pressure to provide a plug. The plug was transferred on top of a column packed with silica gel and was eluted with hexane:ethyl acetate as the eluent. Fractions containing the product (TLC) were pooled and evaporated to afford 257 mg (47 %) of **8i** as a bright yellow crystalline powder. TLC *R_f_* = 0.33 (EtOAc:hexane 1:2 and 2 drops NH_4_OH); ^1^H NMR (499 MHz, DMSO-*d*_6_) δ 7.97 (d, *J* = 9.0 Hz, 1H), 7.09 (d, *J* = 5.5 Hz, 1H), 6.49 (dd, *J* = 9.2, 2.6 Hz, 1H), 6.44 (d, *J* = 2.6 Hz, 1H), 2.78 (d, *J* = 4.7 Hz, 3H), 2.53 (s, 3H).

***N*,4-dimethyl-3-nitroaniline (9i)**

4-methyl-3-nitroaniline (1.52 g, 10 mmol) was added to a solution of NaOMe (2.7 g, 50 mmol) in 20 mL of MeOH. The resulting solution was poured into a suspension of paraformaldehyde (420 mg, 14 mmol) in MeOH (10 mL). The reaction mixture was stirred overnight at room temperature. Sodium borohydride (378.3 mg, 10 mmol) was then added, and the reaction mixture was heated at reflux for 2 hours. After completion of the reaction, 1M KOH was added to the reaction mixture and the product was extracted using EtOAc. The combined organic layers were dried over sodium sulfate and silica gel was added. Next, the solvent was removed under reduced pressure to provide a plug. The plug was transferred on top of a column packed with silica gel and was eluted with hexane:ethyl acetate. Fractions containing the product (TLC) were pooled and evaporated to afford 1.5 g (90 %) of **9i** as a bright orange solid. TLC *Rf* = 0.5 (EtOAc:hexane, 1:2); ^1^H NMR (400 MHz, DMSO-*d*_6_) δ 7.22 – 7.12 (m, 1H), 7.05 (ddd, *J* = 6.3, 3.8, 2.3 Hz, 1H), 6.81 (dtt, *J* = 8.5, 4.1, 2.3 Hz, 1H), 6.14 (s, 1H), 2.69 (d, *J* = 4.3 Hz, 3H), 2.36 – 2.31 (m, 3H).

***N*,3,4-trimethylaniline (10i)**

3,4-Dimethylaniline (2 g, 16.5 mmol) was added to a solution of NaOMe (4.45 g, 82.5 mmol) in 20 mL of MeOH. The resulting solution was poured into a suspension of paraformaldehyde (694 mg, 23 mmol) in MeOH (15 mL). The reaction mixture was stirred overnight at room temperature. Sodium borohydride (624 mg, 16.5 mmol) was then added, and the reaction mixture was heated at reflux for 2 hours. After completion of the reaction, 1M KOH was added to the reaction mixture and the product was extracted using EtOAc. The combined organic layers were dried over sodium sulfate and silica gel was added. Next, the solvent was removed under reduced pressure to provide a plug. The plug was transferred on top of a column packed with silica gel and was eluted with hexane:ethyl acetate. Fractions containing the product (TLC) were pooled and evaporated to afford 660 mg (30 %) of **10i** as an orange liquid. TLC *Rf* = 0.6 (EtOAc:hexane, 1:1); ^1^H NMR (400 MHz, DMSO-*d*_6_) δ 6.84 (dd, *J* = 8.3, 3.8 Hz, 1H), 6.37 (d, *J* = 2.6 Hz, 1H), 6.28 (dt, *J* = 6.8, 3.3 Hz, 1H), 5.26 (s, 1H), 2.63 (d, *J* = 4.1 Hz, 3H), 2.12 (d, *J* = 3.9 Hz, 3H), 2.07 (d, *J* = 4.0 Hz, 3H).

***N*-methyl-2,3-dihydro-1*H*-inden-5-amine (11i)**

2,3-dihydro-1*H*-inden-5-amine (1 g, 7.51 mmol) was added to a solution of NaOMe (2 g, 37.54 mmol) in 20 mL MeOH. The resulting solution was poured into a suspension of paraformaldehyde (316 mg, 10.51 mmol) in MeOH (20 mL). The reaction mixture was stirred overnight at room temperature. Sodium borohydride (284 mg, 7.51 mmol) was then added, and the reaction mixture was heated at reflux for 5 hours. After cooling, silica gel was added to the reaction mixture and the solvent was removed under reduced pressure to provide a plug. The plug was transferred on top of a column packed with silica gel and was eluted with hexane:ethyl acetate as the eluent. Fractions containing the product (TLC) were pooled and evaporated to afford 612 mg (55 %) of **11i** as a dark brown liquid. TLC *R_f_* = 0.41 (EtOAc:hexane 1:4 and 2 drops NH_4_OH); ^1^H NMR (499 MHz, DMSO-*d*_6_) δ 6.92 (d, *J* = 8.0 Hz, 1H), 6.41 (d, *J* = 2.2 Hz, 1H), 6.31 (dd, *J* = 8.0, 2.2 Hz, 1H), 5.36 (s, 1H), 2.71 (dt, *J* = 19.5, 7.3 Hz, 4H), 2.63 (s, 3H), 1.94 (p, *J* = 7.4 Hz, 2H).

***N*-methyl-5,6,7,8-tetrahydronaphthalen-2-amine (12i)**

5,6,7,8-Tetrahydronaphthalen-2-amine (2 g, 13.58 mmol; A453457, Ambeed) was added to a solution of NaOMe (3.67 g, 67.9 mmol) in 20 mL of MeOH. The resulting solution was poured into a suspension of paraformaldehyde (571 mg, 19 mmol) in MeOH (20 mL). The reaction mixture was stirred overnight at room temperature. Sodium borohydride was then added, and the reaction mixture was heated at reflux for 2 hours. After completion of the reaction, 1M KOH was added to the reaction mixture and the product was extracted using EtOAc. The combined organic layers were dried over sodium sulfate and silica gel was added. Next, the solvent was removed under reduced pressure to provide a plug. The plug was transferred on top of a column packed with silica gel and was eluted with hexane:ethyl acetate. Fractions containing the product (TLC) were pooled and evaporated to afford 875 mg (40 %) of **12i** as an orange liquid. TLC *Rf* = 0.6 (EtOAc:hexane, 1:2); ^1^H NMR (400 MHz, DMSO-*d*_6_) δ 6.75 (d, *J* = 8.1 Hz, 1H), 6.32 (dd, *J* = 8.1, 2.5 Hz, 1H), 6.21 (d, *J* = 2.5 Hz, 1H), 5.26 (s, 1H), 2.65 – 2.53 (m, 7H), 1.68 (dq, *J* = 6.7, 2.8 Hz, 4H).

***N*-(4,5-dichloro-9*H*-pyrimido[4,5-*b*]indol-2-yl)pivalamide** (**9**), **1** and **2** (NMR matched with published results) were synthesized according to the published procedure^1, 2^ with modifications to reactions conditions (Scheme 2).

**5-chloro-*N^4^*-(4-ethoxyphenyl)-*N^4^*-methyl-9*H*-pyrimido[4,5-*b*]indole-2,4-diamine (3)**

4-ethoxy-*N*-methylaniline **3i** (127 mg, 3 eq) and 2 drops of concentrated HCl were added to a solution of **9a** (95 mg, 1 eq) in 15 mL of *i*-PrOH. The reaction mixture was heated at 100 ^o^C for 8 hours in a microwave reactor. After cooling to room temperature, 2 mL of 1N sodium hydroxide was added. The reaction was then heated at reflux for 4 hours. After cooling, silica gel was added to the reaction mixture and the solvent was removed under reduced pressure to provide a plug. The plug was transferred on top of a column packed with silica gel and was eluted with hexane:ethyl acetate as the eluent. Fractions containing the product (TLC) were pooled and evaporated to afford 28 mg (27 %) of **3** as a reddish brown solid. TLC *R_f_* = 0.6 (MeOH:DCM, 1:5 and 2 drops NH_4_OH); mp 172.2 °C; ^1^H NMR (500 MHz, DMSO-*d*_6_) δ 11.71 (s, 1H), 7.25 (dd, *J* = 7.9, 0.9 Hz, 1H), 7.16 (td, *J* = 7.9, 0.7 Hz, 1H), 7.01 (dd, *J* = 7.8, 1.0 Hz, 1H), 6.82 – 6.78 (m, 2H), 6.76 – 6.71 (m, 2H), 6.49 (s, 2H), 3.92 (q, 2H), 3.31 (s, 3H), 1.28 (t, *J* = 7.0, 0.7 Hz, 3H). MS (ESI) calculated for C_19_H_19_ClN_5_O [M+H]+, 368.13. Found: 368.13. HPLC analysis: retention time, 13.73 min; peak area, 98.30 %; eluent A, 0.1% formic acid in H_2_O: eluent B, ACN; gradient elution (95% H_2_O to 5% H_2_O) over 30 min with flow rate of 0.5 mL/min and detection at 290 nm; column temperature, rt.


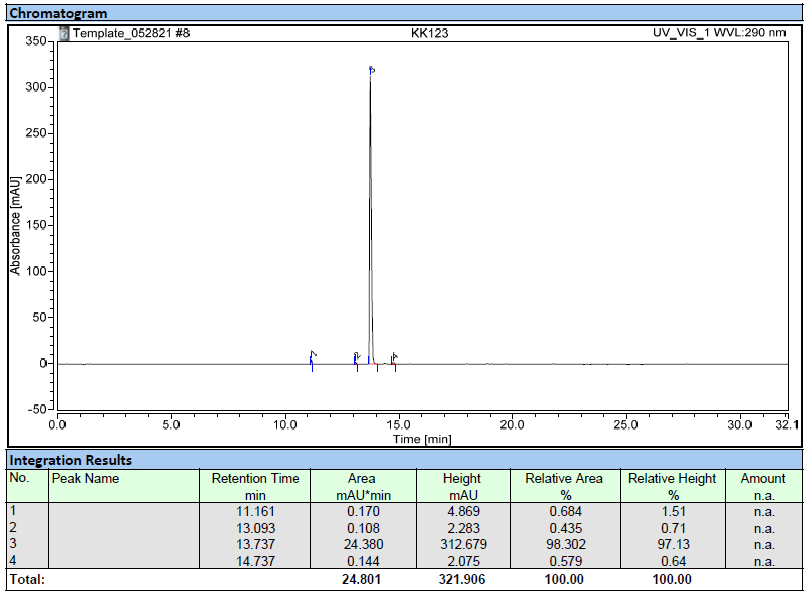


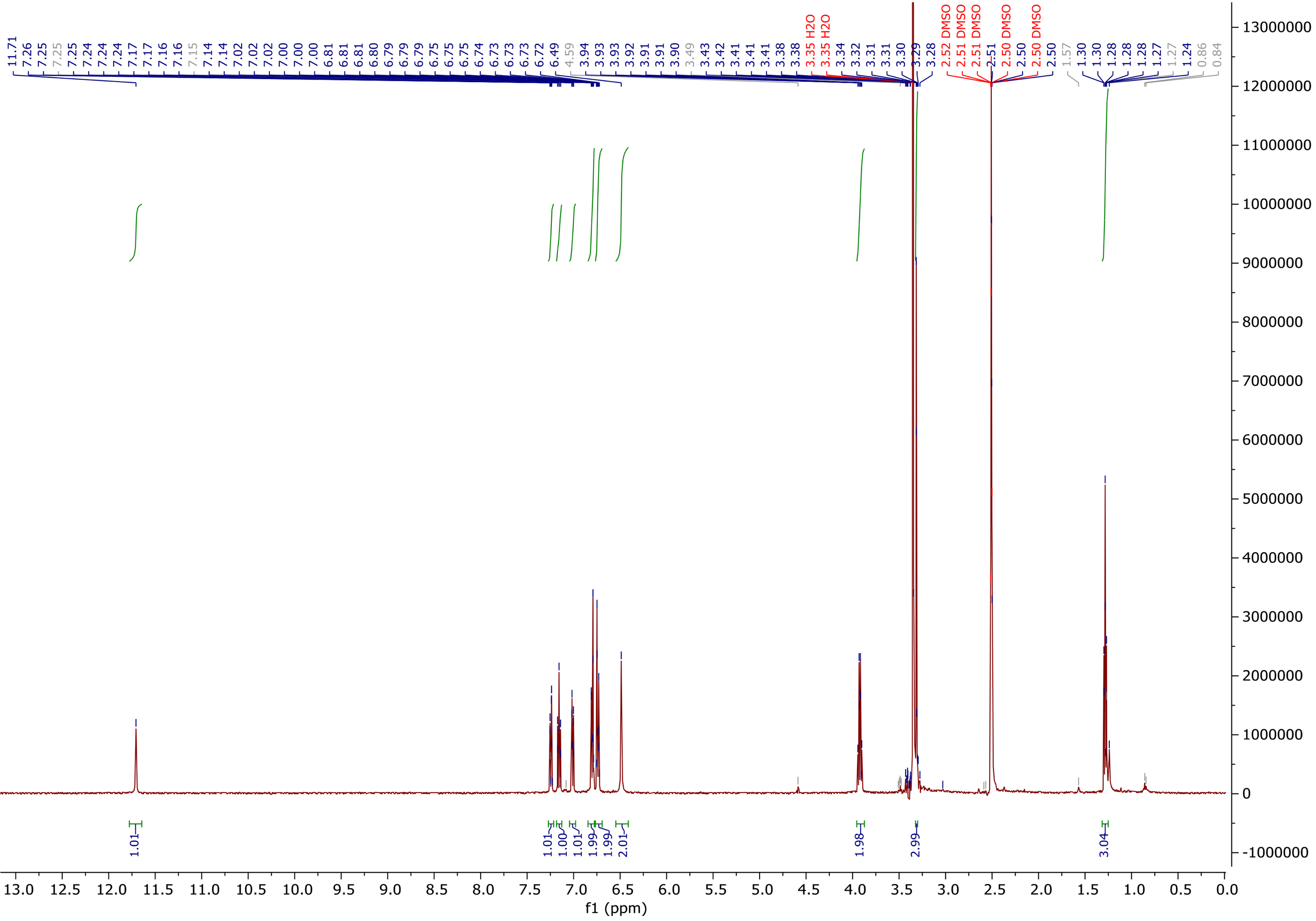


**5-chloro-*N^4^*-(4-methoxy-3-methylphenyl)- *N^4^*-methyl-9*H*-pyrimido[4,5-*b*]indole-2,4-diamine (4)**

4-methoxy-*N*,3-dimethylaniline **4i** (133 mg, 0.88 mmol) and 2 drops of concentrated HCl were added to a solution of **9a** (150 mg, 0.44 mmol) in 2 mL of *i*-propanol in a microwave vial. The reaction mixture was heated at 80 °C in a microwave reactor for 7 hours. The solvent was then evaporated and the resulting residue (150 mg) was dissolved in *i*-propanol (10 mL), and 7 mL of 1N sodium hydroxide solution was added. The reaction was then heated at reflux for 4 hours. Silica gel was added to the reaction mixture, at four times the weight of the reaction mixture, and the solvent was removed under reduced pressure to provide a plug. The plug was transferred on top of a column packed with silica gel (twenty times the weight of plug) and was eluted with DCM:MeOH as the eluent. Fractions containing the product (TLC) were pooled and evaporated to afford 60 mg (37%) of **4** as a pale-yellow powder. TLC *Rf* = 0.42 (MeOH:DCM, 1:10); mp 212 - 213 °C; ^1^H NMR (400 MHz, DMSO-*d*_6_) δ 11.70 (s, 1H), 7.25 (dt, *J* = 7.9, 1.1 Hz, 1H), 7.17 (td, *J* = 7.9, 1.0 Hz, 1H), 7.03 (dd, *J* = 7.8, 1.0 Hz, 1H), 6.80 – 6.71 (m, 2H), 6.64 (dd, *J* = 8.8, 2.9 Hz, 1H), 6.50 – 6.43 (m, 2H), 3.70 (d, *J* = 1.0 Hz, 3H), 3.28 (d, *J* = 1.0 Hz, 3H), 2.06 (s, 3H). MS (ESI) calculated for C_19_H_19_ClN_5_O [M+H]^+^, 368.12. Found: 368.3. HPLC purity 99.87 % (350 nm).

**5-chloro-*N^4^*-methyl-*N^4^*-(4-(methylthio)phenyl)-9*H*-pyrimido[4,5-*b*]indole-2,4-diamine (5)**

*N*-methyl-4-(methylthio)aniline **5i** (271 mg, 1.77 mmol) and 2 drops of concentrated HCl were added to a solution of **9a** (200 mg, 0.59 mmol) in 30 mL of *i*-propanol. The reaction mixture was heated at reflux for 72 hours and cooled to room temperature. The solvent was then evaporated to obtain *N*-(5-chloro-4-((4-(methylthio)phenyl)amino)-9*H*-pyrimido[4,5-*b*]indol-2-yl)pivalamide a yellow solid (150 mg, 56%) which was carried to the next step without further characterization. The yellow solid (130 mg, 0.28 mmol) was dissolved in *i*-propanol (10 mL), and 4 mL of 1N sodium hydroxide solution was added. The reaction was then heated overnight at reflux. Silica gel was added to the reaction mixture, at four times the weight of the reaction mixture, and the solvent was removed under reduced pressure to provide a plug. The plug was transferred on top of a column packed with silica gel (twenty times the weight of plug) and was eluted with hexane:ethyl acetate as the eluent. Fractions containing the product (TLC) were pooled and evaporated to afford 35 mg (34%) of **5** as a yellow powder. TLC *Rf* = 0.30 (MeOH:DCM, 1:20); mp 210 – 211 °C; ^1^H NMR (400 MHz, DMSO-*d*_6_) δ 11.82 (s, 1H), 7.29 (d, *J* = 7.9 Hz, 1H), 7.20 (t, *J* = 7.9 Hz, 1H), 7.11 (d, *J* = 8.3 Hz, 2H), 7.04 (d, *J* = 7.7 Hz, 1H), 6.76 (d, *J* = 8.4 Hz, 2H), 6.59 (s, 2H), 3.30 (s, 3H), 2.38 (s, 3H). MS (ESI) calculated for C_18_H_17_ClN_5_S [M+H]^+^, 370.08. Found: 369.9. HPLC purity 96.58 % (350 nm).

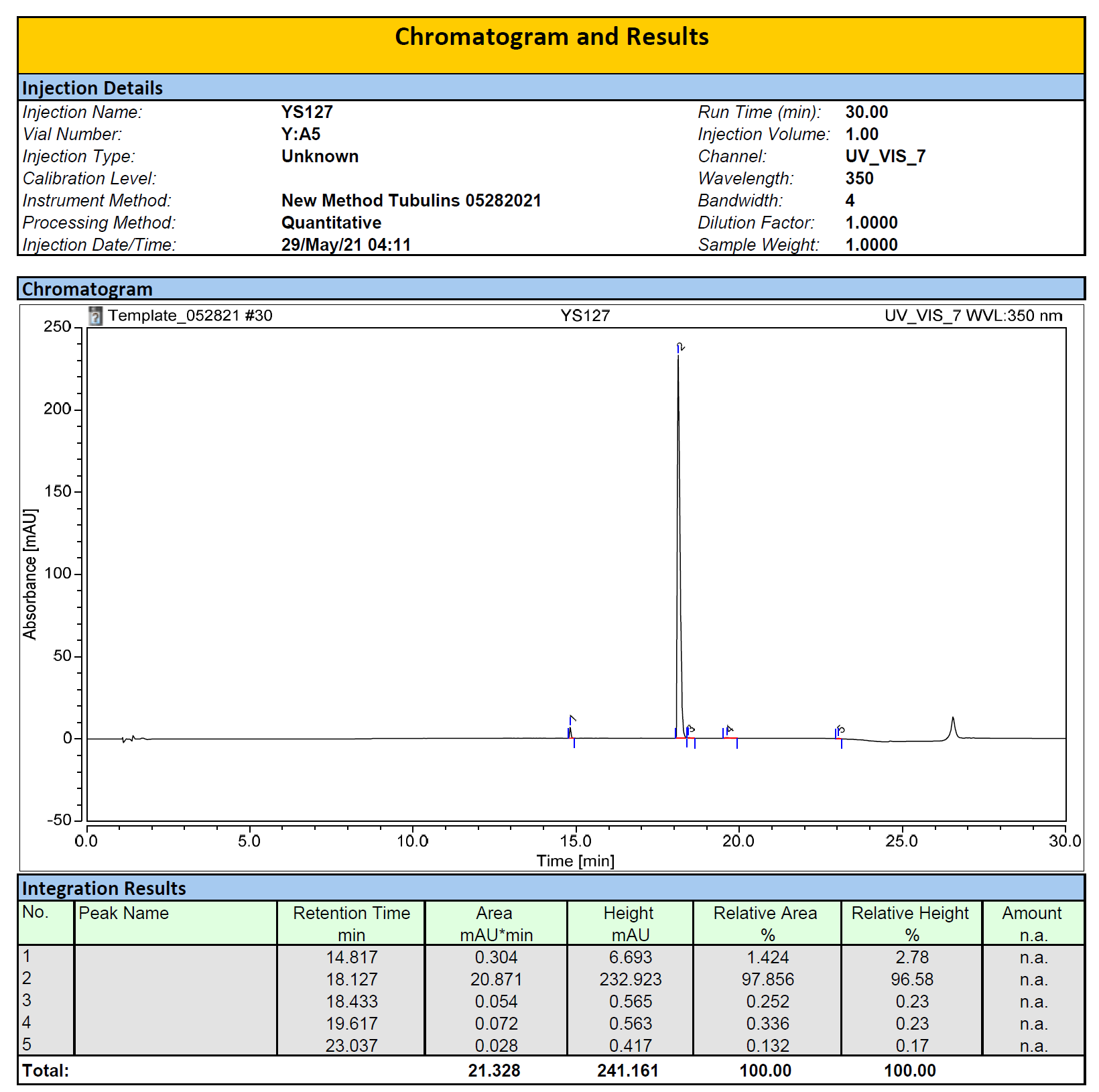


***N^4^*-(benzofuran-5-yl)-5-chloro-*N^4^*-methyl-9*H*-pyrimido[4,5-*b*]indole-2,4-diamine (6)**

*N*-methylbenzofuran-5-amine **6i** (148 mg, 1.01 mmol) and 2 drops of concentrated HCl were added to a solution of **9a** (113 mg, 0.33 mmol) in 15 mL of *n*-butanol. The reaction mixture was heated at 150 ^o^C for 4 hours in a microwave reactor. After cooling to room temperature, 2 mL of 1N sodium hydroxide were added. The reaction was then heated at reflux for 4 hours. After cooling, silica gel was added to the reaction mixture and the solvent was removed under reduced pressure to provide a plug. The plug was transferred on top of a column packed with silica gel and was eluted with hexane:ethyl acetate as the eluent. Fractions containing the product (TLC) were pooled and evaporated to afford 31 mg (25 %) of **6** as a buff-colored powder. TLC *Rf* = 0.54 (MeOH:DCM, 1:5 and 2 drops NH_4_OH); mp 200 °C; ^1^H NMR (500 MHz, DMSO-*d*_6_) δ 11.77 (s, 1H), 7.88 (d, *J* = 7.9 Hz, 1H), 7.38 (dt, *J* = 7.9 Hz, 1H), 7.27 (dd, *J* = 7.8 Hz, 1H), 7.17 (t, *J* = 7.8 Hz, 1H), 7.10 (d, *J* = 7.7 Hz, 1H), 7.02 (dd, *J* = 7.7 Hz, 1H), 6.89 (dd, *J* = 8.2 Hz, 1H), 6.82 (dd, *J* = 8.2 Hz, 1H), 6.53 (s, 2H), 3.41 (s, 3H). MS (ESI) calculated for C_19_H_15_ClN_5_O [M+H]^+^, 364.10. Found: 364.40. HPLC analysis: retention time, 13.44 min; peak area, 95.67 %; eluent A, 0.1% formic acid in H_2_O: eluent B, ACN; gradient elution (95% H_2_O to 5% H_2_O) over 30 min with flow rate of 0.5 mL/min and detection at 290 nm; column temperature, rt.


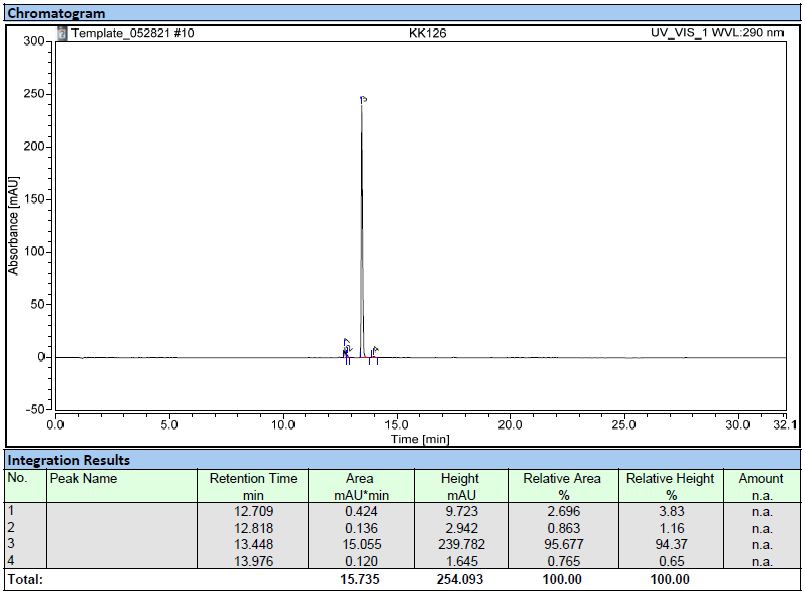


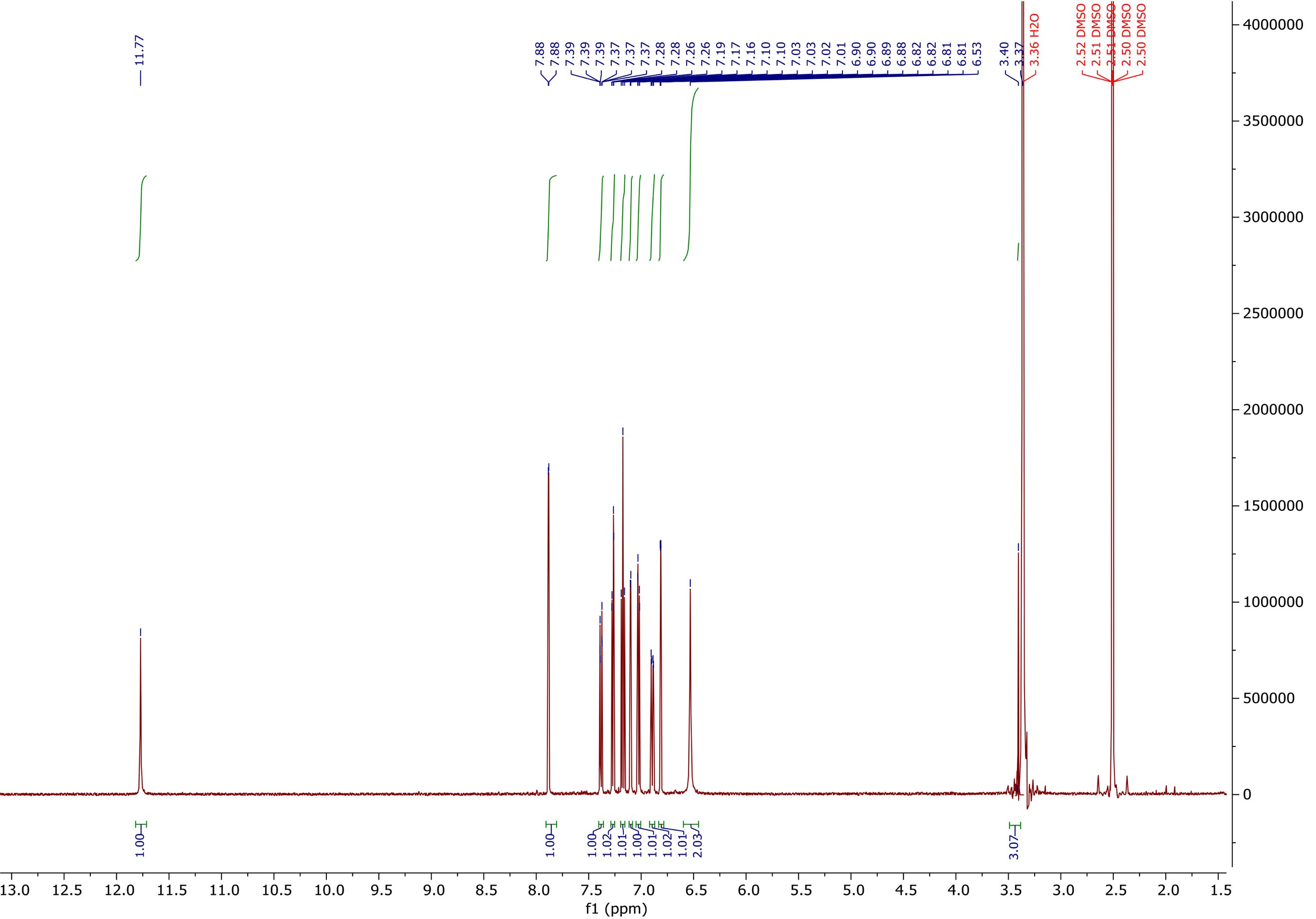


***N^4^*-(benzo[*b*]thiophen-5-yl)-5-chloro-*N^4^*-methyl-9*H*-pyrimido[4,5-*b*]indole-2,4-diamine (7)**

N-methylbenzo[*b*]thiophen-5-amine (**7i**) (96 mg, 0.58 mmol) and 2 drops of concentrated HCl were added to a solution of **9a** (132 mg, 0.39 mmol) in 15 mL of *n*-butanol. The reaction mixture was heated at 150 ^o^C for 4 hours in a microwave reactor. After cooling to room temperature, 2 mL of 1N sodium hydroxide were added. The reaction was then heated at reflux for 4 hours. After cooling, silica gel was added to the reaction mixture and the solvent was removed under reduced pressure to provide a plug. The plug was transferred on top of a column packed with silica gel and was eluted with hexane:ethyl acetate as the eluent. Fractions containing the product (TLC) were pooled and evaporated to afford 27 mg (18 %) of **7** as an off-white powder. TLC *R_f_* = 0.57 (MeOH:DCM, 1:5 and 2 drops NH_4_OH); mp 270.8 °C; ^1^H NMR (500 MHz, DMSO-*d*_6_) δ 11.82 (s, 1H), 7.72 (d, *J* = 8.8 Hz, 1H), 7.65 (dd, *J* = 5.3, 1.2 Hz, 1H), 7.30 (ddd, *J* = 9.1, 6.3, 1.7 Hz, 3H), 7.19 (td, *J* = 7.9, 1.1 Hz, 1H), 7.03 (dd, *J* = 7.9, 1.0 Hz, 1H), 6.95 (dd, *J* = 8.9, 2.4 Hz, 1H), 6.59 (s, 2H), 3.38 (s, 3H). MS (ESI) calculated for C_19_H_15_ClN_5_S [M+H]+, 380.07. Found: 380.06. HPLC analysis: retention time, 14.66 min; peak area, 99.81 %; eluent A, 0.1% formic acid in H_2_O: eluent B, ACN; gradient elution (95% H_2_O to 5% H_2_O) over 30 min with flow rate of 0.5 mL/min and detection at 290 nm; column temperature, rt.


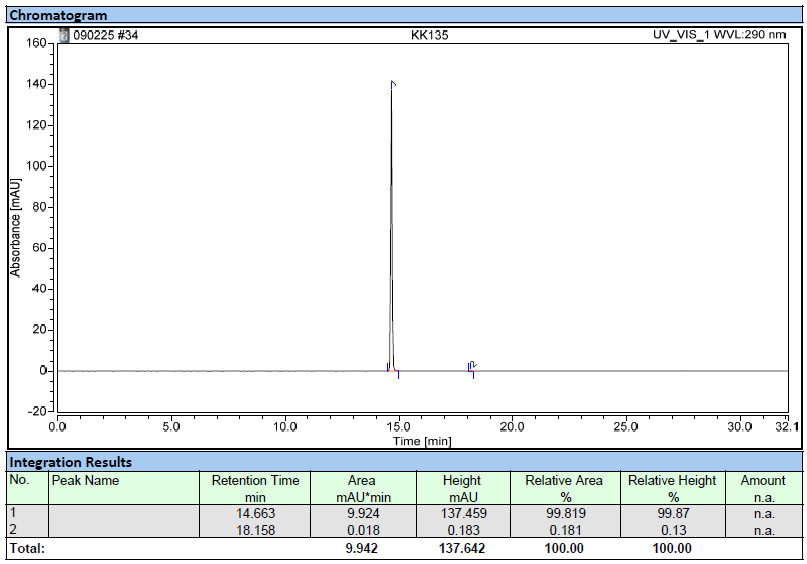


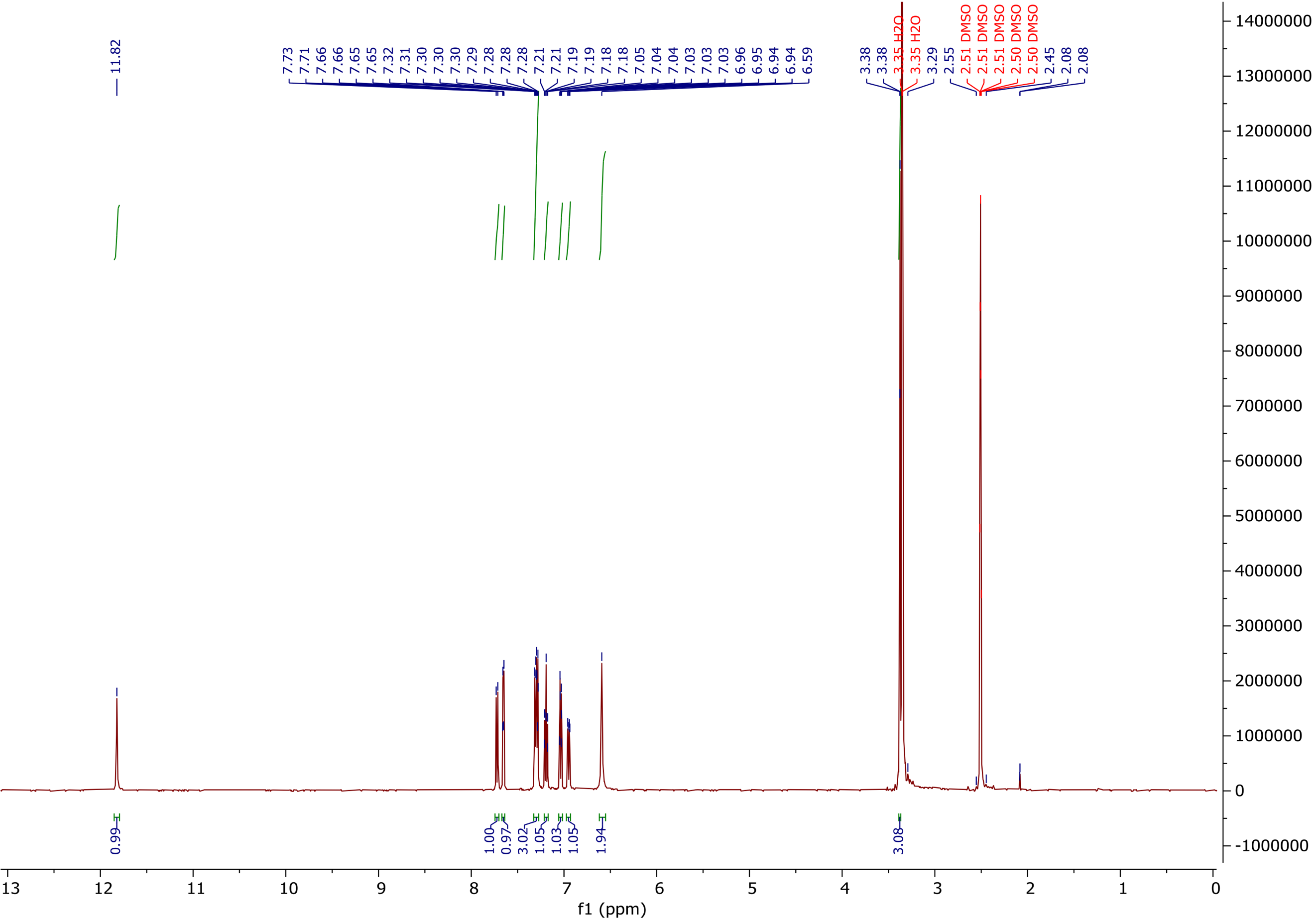


**5-chloro-*N^4^*-methyl-*N^4^*-(3-methyl-4-nitrophenyl)-9*H*-pyrimido[4,5-*b*]indole-2,4-diamine (8):** *N*,3-dimethyl-4-nitroaniline (**8i**) (86 mg, 0.51 mmol) and 2 drops of concentrated HCl were added to a solution of **9a** (145 mg, 0.43 mmol) in 15 mL of anhydrous acetonitrile. The reaction mixture was heated at 100 ^o^C for 20 hours in a microwave reactor. After cooling to room temperature, 2 mL of 1N sodium hydroxide were added. The reaction was then heated at reflux for 4 hours. After cooling, silica gel was added to the reaction mixture and the solvent was removed under reduced pressure to provide a plug. The plug was transferred on top of a column packed with silica gel and was eluted with hexane:ethyl acetate as the eluent. Fractions containing the product (TLC) were pooled and evaporated to afford 70 mg (43 %) of **8** as a bright yellow crystalline powder. TLC *R_f_* = 0.58 (MeOH:DCM, 1:5 and 2 drops NH_4_OH); mp 271.1 °C (decomposed); ^1^H NMR (400 MHz, Acetone-*d*_6_) δ 11.03 (s, 1H), 7.97 (d, *J* = 9.2 Hz, 1H), 7.42 (dd, *J* = 7.9, 1.0 Hz, 1H), 7.29 (t, *J* = 7.9 Hz, 1H), 7.12 (dd, *J* = 7.9, 1.0 Hz, 1H), 6.77 (d, *J* = 2.9 Hz, 1H), 6.69 (dd, *J* = 9.2, 2.8 Hz, 1H), 6.21 (s, 2H), 3.50 (s, 3H), 2.54 (s, 3H). MS (ESI) calculated for C_18_H_16_ClN_6_O_2_ [M+H]+, 383.10. Found: 383.09. HPLC analysis: retention time, 15.85 min; peak area, 98.04 %; eluent A, 0.1% formic acid in H_2_O: eluent B, ACN; gradient elution (95% H_2_O to 5% H_2_O) over 30 min with flow rate of 0.5 mL/min and detection at 290 nm; column temperature, rt.


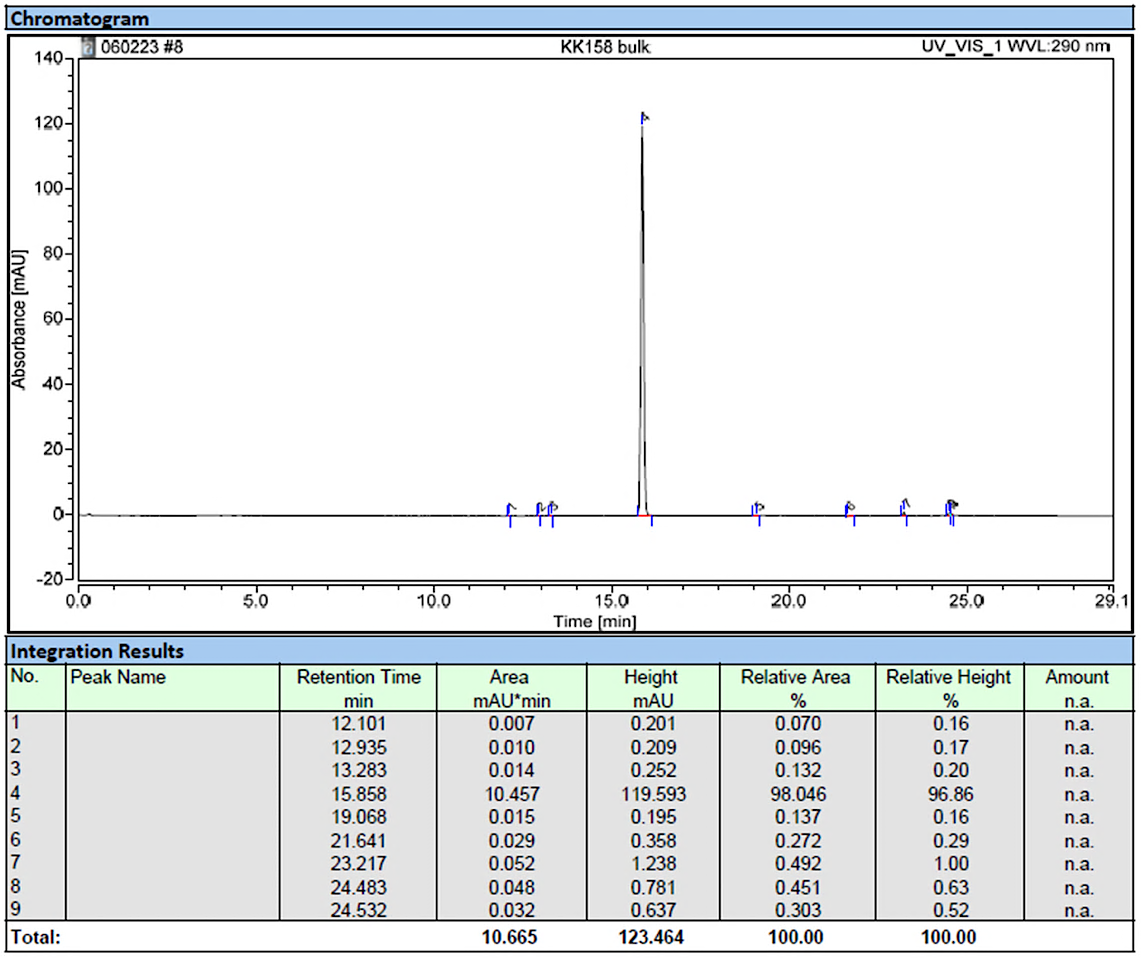


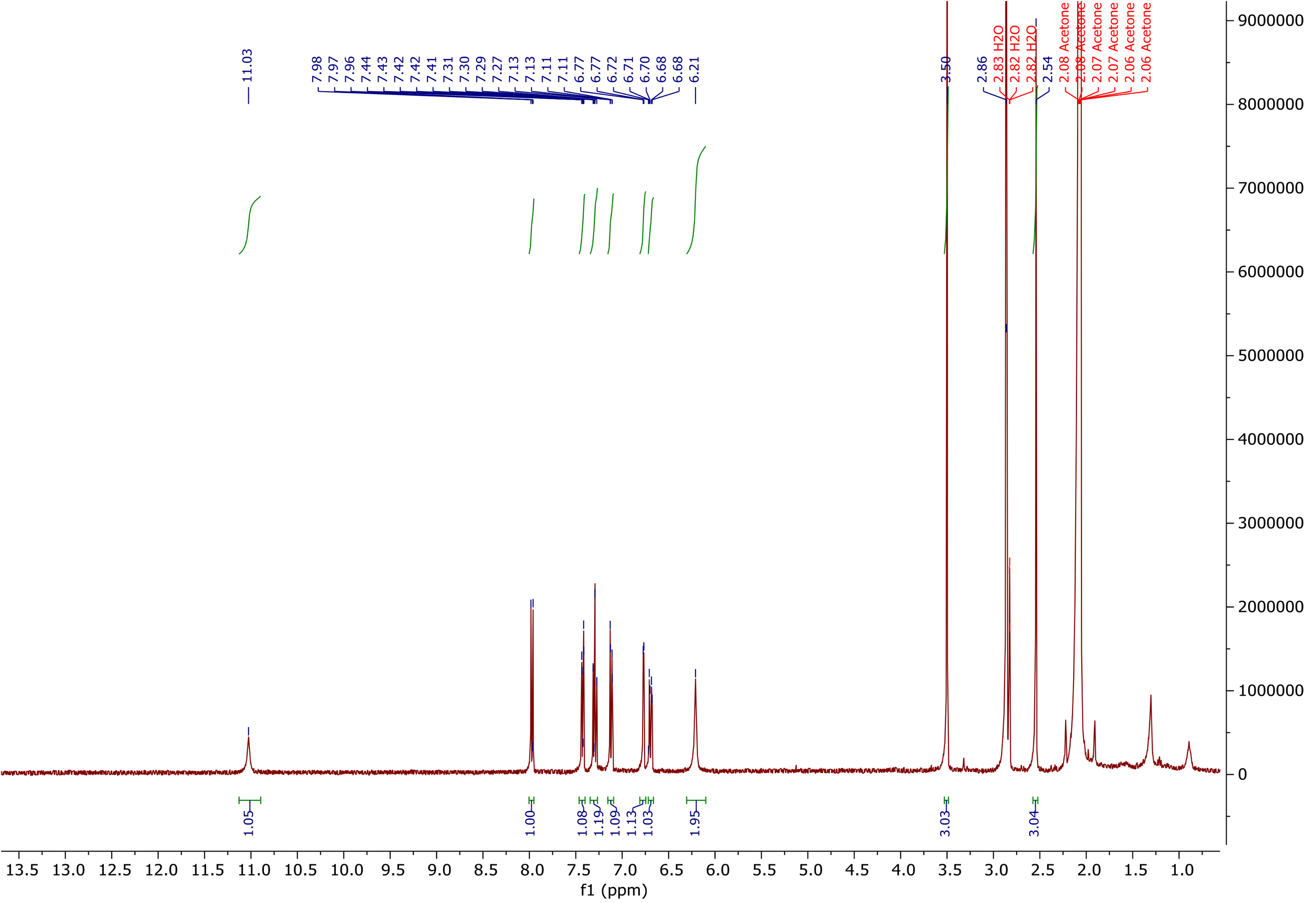


**5-chloro-*N^4^*-methyl- *N^4^*-(4-methyl-3-nitrophenyl)-9*H*-pyrimido[4,5-*b*]indole-2,4-diamine (9)**

*N*,4-dimethyl-3-nitroaniline **9i** (146 mg, 0.88 mmol) and 2 drops of concentrated HCl were added to a solution of **9a** (150 mg, 0.44 mmol) in 3 mL of *i*-propanol in a microwave vial. The reaction mixture was heated at 80 °C in a microwave reactor for 12 hours. The solvent was then evaporated, and the resulting yellow residue was dissolved in *i*-propanol (5 mL), and 2 mL of 1N sodium hydroxide solution was added. The reaction was then heated at reflux for 4 hours. Silica gel was added to the reaction mixture, at four times the weight of the reaction mixture, and the solvent was removed under reduced pressure to provide a plug. The plug was transferred on top of a column packed with silica gel (twenty times the weight of plug) and was eluted with DCM:MeOH as the eluent. Fractions containing the product (TLC) were pooled and evaporated to afford 90 mg (53%) of **9** as a yellow powder. TLC *Rf* = 0.42 (MeOH:DCM, 1:10); mp 285 - 286 °C; ^1^H NMR (400 MHz, DMSO-*d*_6_) δ 11.93 (s, 1H), 7.35 – 7.30 (m, 2H), 7.24 (t, *J* = 7.9 Hz, 2H), 7.08 (d, *J* = 7.8 Hz, 1H), 7.01 (dd, *J* = 8.4, 2.7 Hz, 1H), 6.74 (s, 2H), 2.40 (s, 3H). MS (ESI) calculated for C_28_H_16_ClN_6_O_2_ [M+H]^+^, 383.09. Found: 383.0. HPLC purity 99.94 % (350 nm).

**5-chloro-*N^4^*-(3,4-dimethylphenyl)- *N^4^*-methyl-9*H*-pyrimido[4,5-*b*]indole-2,4-diamine (10)**

*N*,3,4-trimethylaniline **10i** (240 mg, 1.78 mmol) and 2 drops of concentrated HCl were added to a solution of **9a** (200 mg, 0.59 mmol) in 30 mL of *i*-propanol. The reaction mixture was heated at reflux for 6 days and cooled to room temperature. The solvent was then evaporated to obtain *N*-(5-chloro-4-((3,4-dimethylphenyl)(methyl)amino)-9*H*-pyrimido[4,5-*b*]indol-2-yl)pivalamide which was carried to the next step without further characterization. The crude solid was dissolved in *i*-propanol (10 mL), and 4 mL of 1N sodium hydroxide solution was added. The reaction was then heated overnight at reflux. Silica gel was added to the reaction mixture, at four times the weight of the reaction mixture, and the solvent was removed under reduced pressure to provide a plug. The plug was transferred on top of a column packed with silica gel (twenty times the weight of plug) and was eluted with hexane:ethyl acetate as the eluent. Fractions containing the product (TLC) were pooled and evaporated to afford 95 mg (46%) of **10** as a yellow powder. TLC *Rf* = 0.25 (MeOH:DCM, 1:20) mpt; 200 °C. ^1^H NMR (400 MHz, DMSO-*d*_6_) δ 11.76 (s, 1H), 7.27 (d, *J* = 7.9 Hz, 1H), 7.18 (t, *J* = 7.9 Hz, 1H), 7.04 (d, *J* = 7.8 Hz, 1H), 6.89 (d, *J* = 8.2 Hz, 1H), 6.69 (d, *J* = 2.5 Hz, 1H), 6.54 (s, 3H), 3.28 (s, 3H), 2.11 (s, 6H). MS (ESI) calculated for C_19_H_19_ClN_5_ [M+H]^+^, 352.13. Found: 352.3. HPLC purity 99.2 % (350 nm).


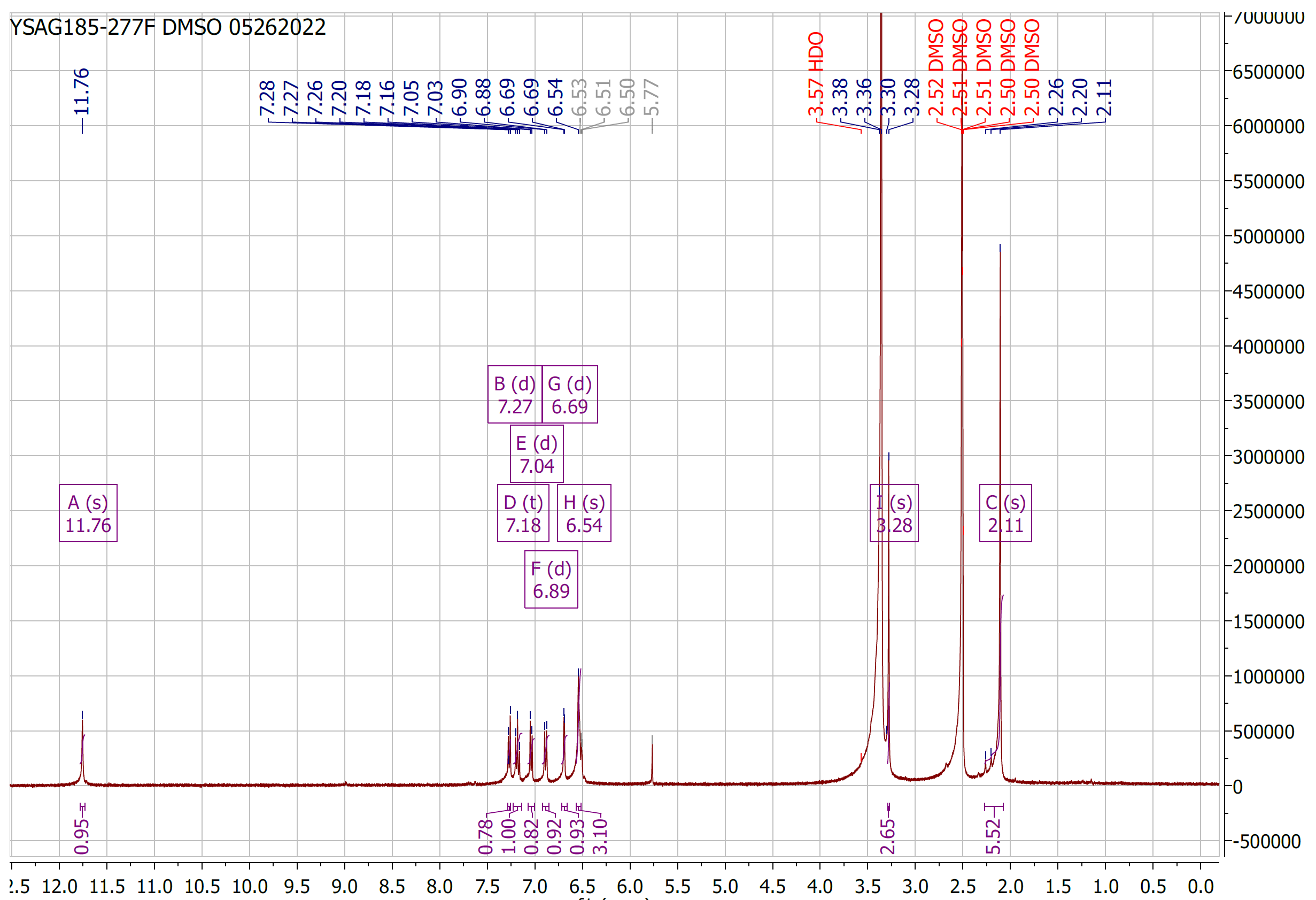

**5-chloro-*N^4^*-(2,3-dihydro-1*H*-inden-5-yl)-*N^4^*-methyl-9*H*-pyrimido[4,5-*b*]indole-2,4-diamine (11)**

*N*-methyl-2,3-dihydro-1*H*-inden-5-amine (**11i**) (175 mg, 1.19 mmol) and 2 drops of concentrated HCl were added to a solution of **9a** (200 mg, 0.59 mmol) in 15 mL of anhydrous acetonitrile. The reaction mixture was heated at 100 ^o^C for 4 hours in a microwave reactor. After cooling to room temperature, 2 mL of 1N sodium hydroxide were added. The reaction was then heated at reflux for 4 hours. After cooling, silica gel was added to the reaction mixture and the solvent was removed under reduced pressure to provide a plug. The plug was transferred on top of a column packed with silica gel and was eluted with hexane:ethyl acetate as the eluent. Fractions containing the product (TLC) were pooled and evaporated to afford 42 mg (20 %) of **11** as a buff-colored crystalline powder. TLC *R_f_* = 0.7 (MeOH:DCM, 1:5 and 2 drops NH_4_OH); mp 222.1 °C; ^1^H NMR (500 MHz, DMSO-*d*_6_) δ 12.42 (d, *J* = 1.9 Hz, 1H), 7.41 (dq, *J* = 8.1, 1.0 Hz, 1H), 7.35 – 7.30 (m, 1H), 7.13 (dq, *J* = 7.8, 1.0 Hz, 1H), 6.98 – 6.93 (m, 1H), 6.78 (d, *J* = 2.3 Hz, 1H), 6.57 (dd, *J* = 8.2, 2.3 Hz, 1H), 6.40 (s, 2H), 3.46 (s, 3H), 2.78 – 2.69 (m, 4H), 2.00 – 1.91 (m, 2H). MS (ESI) calculated for C_20_H_19_ClN_5_ [M+H]+, 364.13. Found: 364.15. HPLC analysis: retention time, 20.53 min; peak area, 97.50 %; eluent A, 0.1% formic acid in H_2_O: eluent B, ACN; gradient elution (95% H_2_O to 5% H_2_O) over 30 min with flow rate of 0.5 mL/min and detection at 290 nm; column temperature, rt.


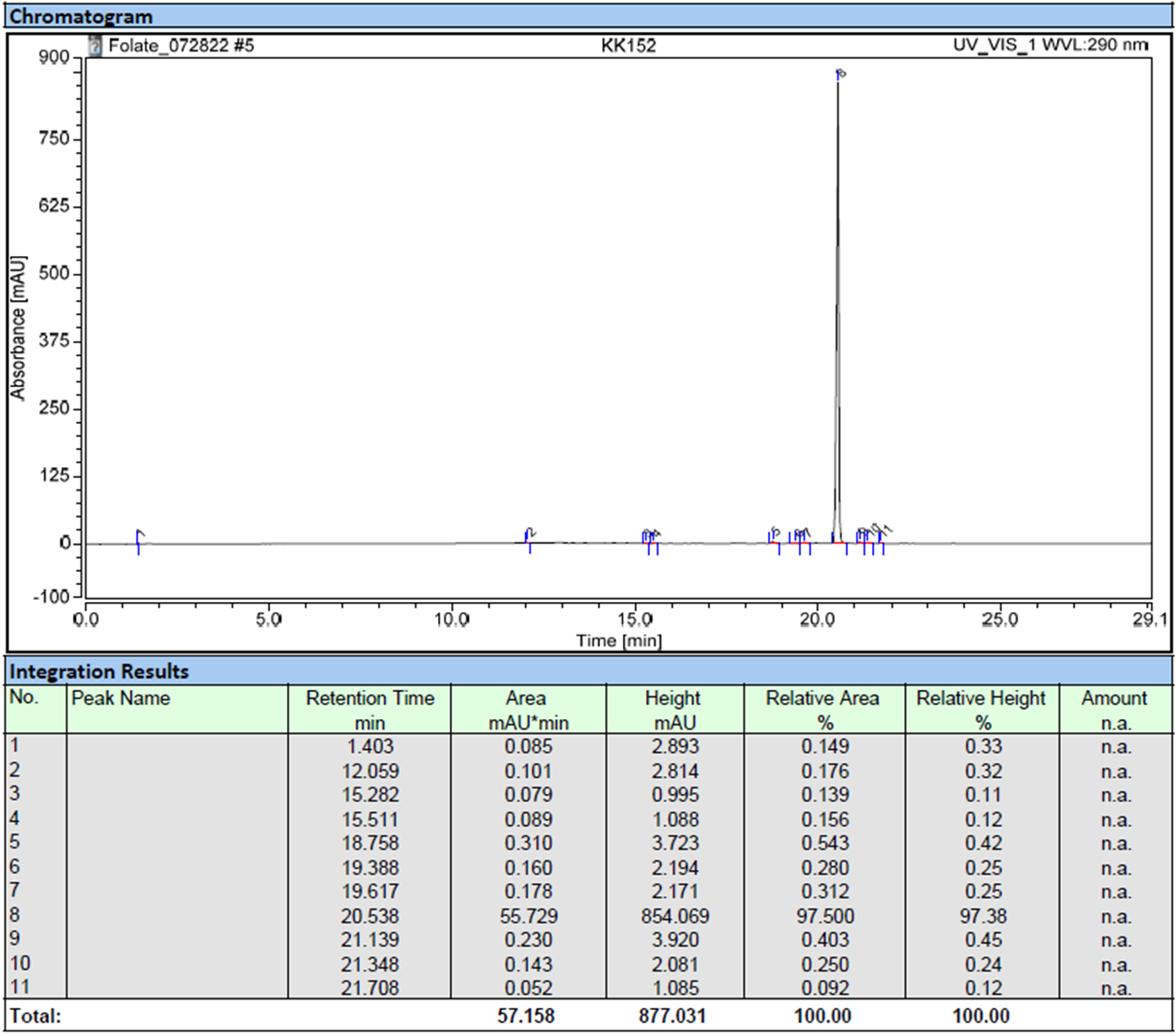


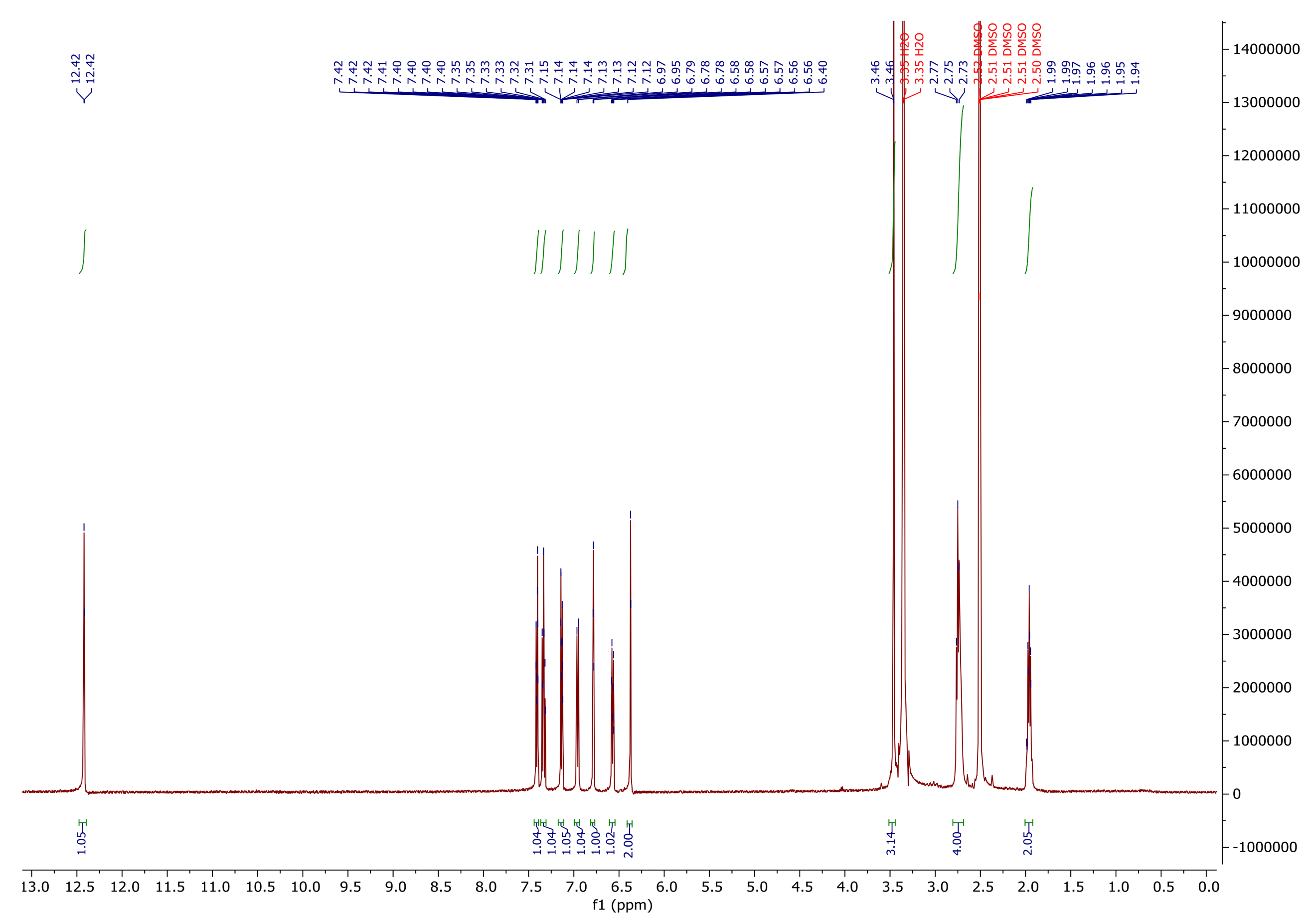


**5-Chloro-*N^4^*-methyl-*N^4^*-(5,6,7,8-tetrahydronaphthalen-2-yl)-9*H*-pyrimido[4,5-*b*]indole-2,4-diamine (12)**

*N-*methyl-5,6,7,8-tetrahydronaphthalen-2-amine **12i** (358 mg, 2.22 mmol) and 2 drops of concentrated HCl were added to a solution of **9a** (250 mg, 0.74 mmol) in 50 mL of *i*-propanol. The reaction mixture was heated at reflux for 120 hours and cooled to room temperature. The solvent was then evaporated to obtain a dark brown colored solid which was dissolved in *i*-propanol (10 mL), and 5 mL of 1N sodium hydroxide solution was added. The reaction was then heated at reflux for 4 hours. Silica gel was added to the reaction mixture, at four times the weight of the reaction mixture, and the solvent was removed under reduced pressure to provide a plug. The plug was transferred on top of a column packed with silica gel (twenty times the weight of plug) and was eluted with hexane:ethyl acetate as the eluent. Fractions containing the product (TLC) were pooled and evaporated to afford 84 mg (30 %) of **12** as a light brown powder. TLC *Rf* = 0.15 (EtOAc:hexane, 1:2); mp 192 - 194 °C; ^1^H NMR (400 MHz, DMSO-*d*_6_) δ 11.77 (s, 1H), 7.28 (d, *J* = 7.9 Hz, 1H), 7.19 (t, *J* = 7.9 Hz, 1H), 7.05 (d, *J* = 7.8 Hz, 1H), 6.81 (d, *J* = 8.2 Hz, 1H), 6.56 (t, *J* = 8.9 Hz, 4H), 3.26 (s, 3H), 2.60 (s, 4H), 1.67 (s, 4H). MS (ESI) calculated for C_21_H_21_ClN_5_ [M+H]^+^, 378.88. Found: 378.5. HPLC purity 97.60 % (350 nm).

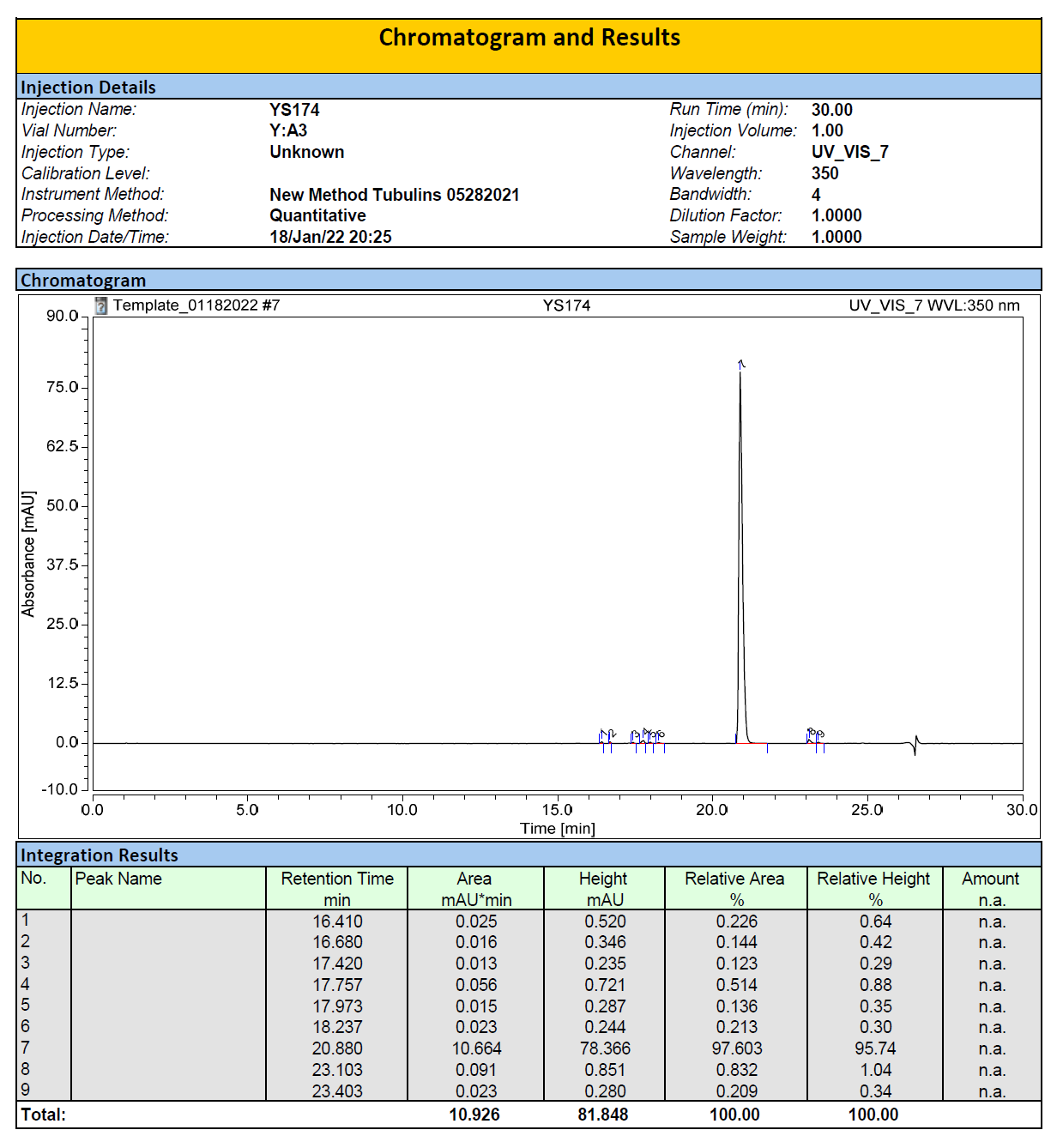


***N^4^*-benzyl-5-chloro-*N^4^*-methyl-9*H*-pyrimido[4,5-*b*]indole-2,4-diamine (13)**

*N-*methyl-1-phenylmethanamine (**13i**) (0.51 mmol) and 2 drops of concentrated HCl were added to a solution of **9a** (0.5 mmol) in 15 mL of anhydrous acetonitrile. The reaction mixture was heated at 100 ^o^C for 8 hours in a microwave reactor. After cooling to room temperature, 2 mL of 1N sodium hydroxide were added. The reaction was then heated at reflux for 4 hours. After cooling, silica gel was added to the reaction mixture and the solvent was removed under reduced pressure to provide a plug. The plug was transferred on top of a column packed with silica gel and was eluted with hexane:ethyl acetate as the eluent. Fractions containing the product (TLC) were pooled and evaporated to afford 70 mg (30 %) of **13** as a yellow powder. TLC *R_f_* = 0.73 (MeOH:DCM, 1:5 and 2 drops NH_4_OH); mp 235 °C. 1H NMR (500 MHz, DMSO) δ 11.58 (s, 1H), 7.29 (d, J = 7.1 Hz, 1H), 7.28 – 7.26 (m, 1H), 7.26 – 7.23 (m, 1H), 7.23 – 7.18 (m, 1H), 7.17 (d, J = 7.9 Hz, 1H), 7.15 (s, 1H), 7.14 (d, J = 5.3 Hz, 1H), 7.13 – 7.10 (m, 1H), 6.35 (s, 2H), 4.68 (s, 2H), 2.83 (s, 3H).MS (ESI) calculated for C_18_H_16_ClN_5_ [M+H]+, 337.81. Found: 337.88.


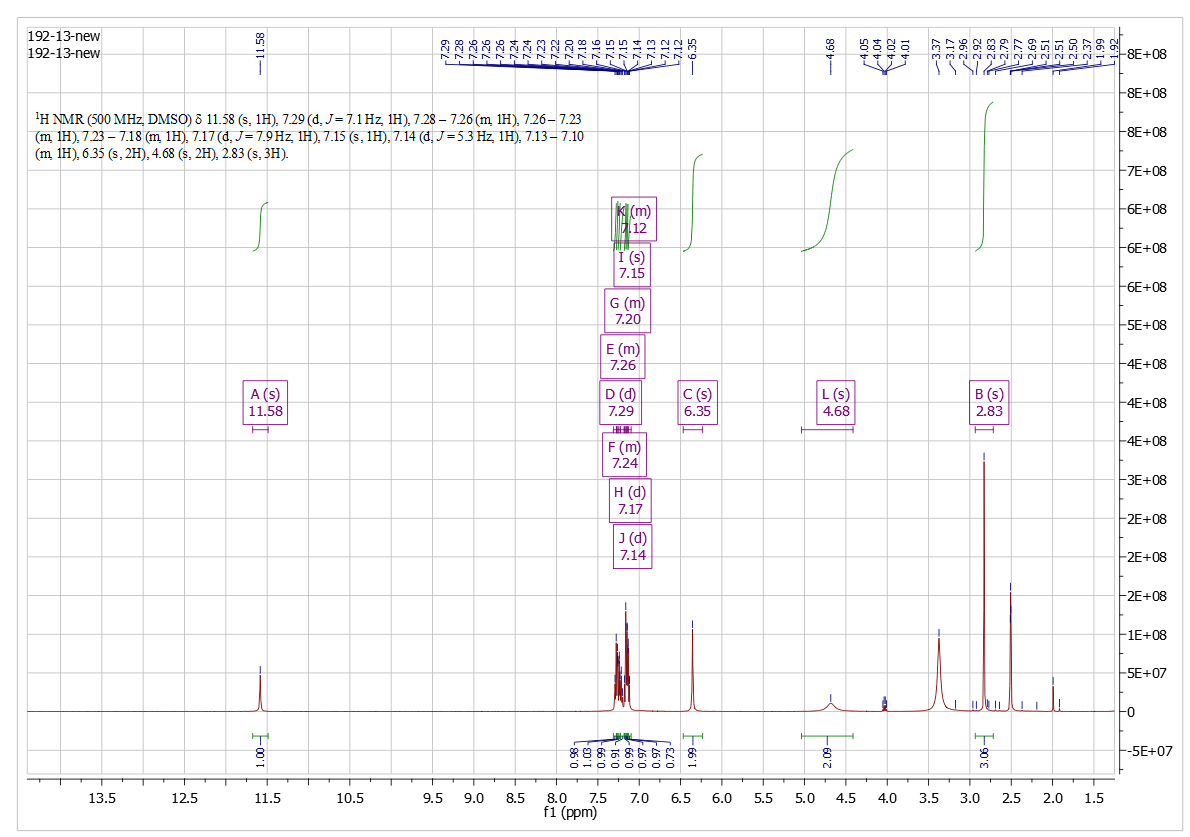


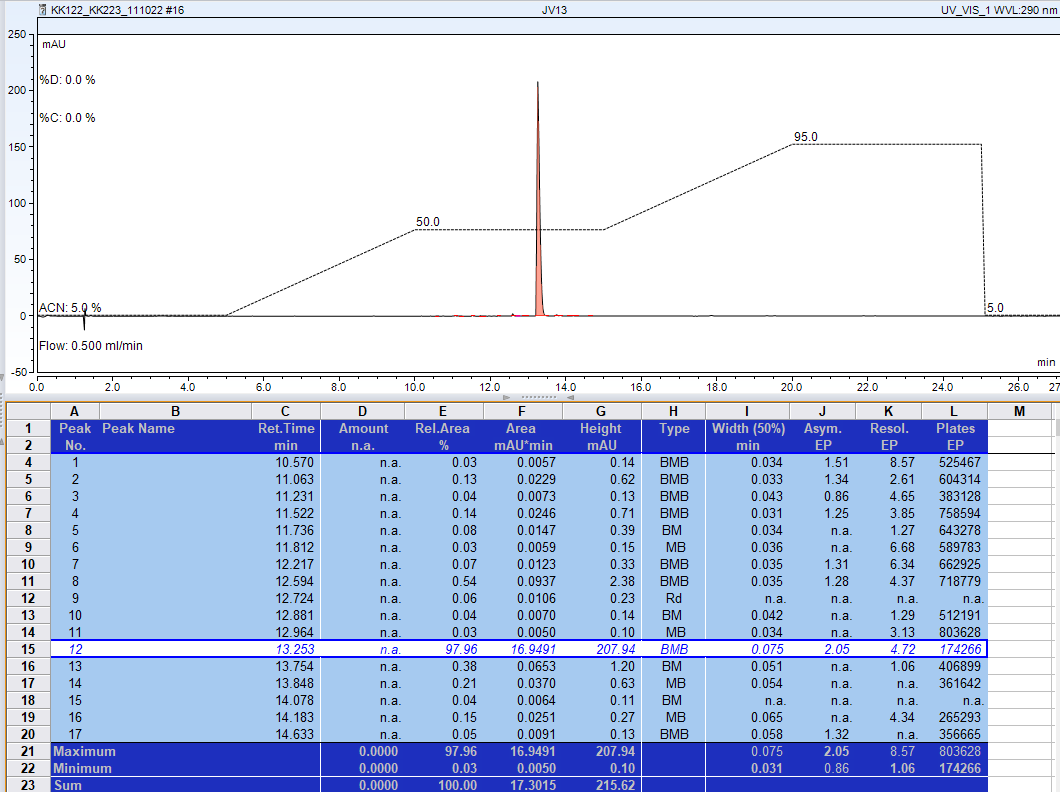


1. *Experimental procedures:*

***Docking protocol:***

Ligands to be docked were prepared using the LigPrep module in Maestro (Schrödinger).^3^ The proteins of interest were prepared using the protein preparation workflow in Maestro. Next, the compounds were docked in the proteins using Maestro's “induced fit” docking module. Default settings were used for all three actions. The docked structures of the compounds were examined for relevant, top scoring docked poses. The selected docked structures were then superimposed using the C-alpha atoms of the protein with Root Mean Square Deviation (RMSD) values < 0.07.

***Preparation of preformed α-synuclein fibrils:***

Preformed α-synuclein fibrils (PFFs) were synthesized and validated as a kind gift from Dr. Kelvin Luk’s lab at the University of Pennsylvania.^4, 5^ This batch of PFFs has been used and described in four recent publications from the Leak lab.^6-9^ Before shipment to Duquesne, the fibrils were evaluated by sedimentation for high MW species, Thioflavin assays for ß-sheets, bicinchoninic protein concentration tests, and endotoxins tests (< 0.11 pg lipopolysaccharides / µg protein), according to established guidelines.^4, 5^ On the day of *in vitro* use, the fibrils were diluted from the stock concentration of 5 μg/μL to 1 μg/μL in neuronal media (ingredients listed below). PFFs were sonicated in a closed tube for 1 hour at 40 kHz in a water bath at room temperature, as described^10-12^ (Bransonic series model M1800, Branson Ultrasonics Corporation).

***Primary hippocampal cultures:***

With Institutional Animal Care and Use Committee (IACUC) approval, primary hippocampal cultures were generated from postnatal day 0-2 rat pups, as described in our prior reports.^8, 13^ Briefly, brains were harvested from Sprague Dawley rat pups and immersed in Hibernate A (HA; BrainBits, LLC.). The hippocampi were dissected and treated with papain (20 U/mL; LS 3126, Worthington Biochemical Corporation) for 30 minutes at 37 ℃ and 5 % CO_2_ to reduce intercellular adhesion. Papain was neutralized with 0.5 U of DNAse solution (DN25, Sigma-Aldrich), and tissues were gently triturated with a 5 mL serological pipette to generate a homogeneous cell suspension. The cell suspensions were filtered through 40 μm cell strainers and then plated in 12-well (353043, Falcon) or 96-well plates (3595, Corning Costar) or 3.5 mm dishes (353001, Falcon Corning), coated with 66.7 μg/mL poly-D-lysine (P0899, Sigma-Aldrich) and 6.7 μg/mL mouse laminin (354232, Corning).

Neurons were plated in Neurobasal-A medium (10888022, ThermoFisher Scientific) with GlutaMAX (35-050-061, ThermoFisher Scientific), penicillin/streptomycin (15-140-122, ThermoFisher Scientific), B27 (17-504-044, ThermoFisher Scientific), and 10 % fetal bovine serum (S11550H, Atlanta Biologicals). Plating media were exchanged for neuronal media (same ingredients but without fetal bovine sera) at 18 hours. Cultures were incubated at 37 °C in 5 % CO_2_ and treated on day-*in-vitro* (DIV) 2 with the appropriate vehicle or test compounds, fibrils, and nocodazole (M1404, Sigma-Aldrich) in a 50 % media exchange. For long-term cultures, 50 % of the media was again refreshed 7 days post-treatment on DIV 9. Treated cell cultures were fixed, or lysates were collected on DIV 12, as described below, unless indicated otherwise. For long-term survival neuronal cultures, the cells were exposed to fibrils and/or treatments from DIV 2 to DIV 12.

***Immunocytochemistry:***

On DIV 12, cells were fixed and permeabilized with a cocktail of 3 % paraformaldehyde (Formal-Fixx, 9990244, ThermoFisher Scientific) and 2 % sucrose in 0.1 M phosphate buffer, with or without the nonionic detergent Triton X-100 at 1 % v/v. Permeabilized cells were blocked using LI-COR Intercept Blocking Buffer PBS (927-70001, LI-COR) and immunostained as described previously.^8, 13^ Plates were imaged and quantified as In-Cell Westerns using an Odyssey imager (LI-COR) and LI-COR ImageStudio software (Ver. 5.2.5), or imaged at higher resolution on an epifluorescence microscope (Olympus IX73, B&B Microscopes). Wells subjected to all staining procedures but with omission of the primary antibody were used for background subtraction calculations in the In-Cell Western analyses.

Automated image analysis on blind-coded images was performed with Olympus cellSens software (Version 2.3). All nuclei were labeled with the pan-nuclear marker Hoechst 33258 (bisBenzimide, 5 μg/mL; B1155, Sigma-Aldrich). For viable cell counts, clusters of Hoechst^+^ nuclei with an area > 500 μm^2^, nuclei with an area < 20 μm^2^, or nuclei with a mean grey intensity (MGI) > 600 arbitrary units (a.u.) in cellSens were excluded *a priori* as evidence of cell death and chromatin condensation.^14^ Nuclei with areas < 20 μm^2^ *and* MGI > 600 a.u. were thus defined as nonviable cells. For quantification of area fractions, cellSens divides the total number of pixels with target staining by the total number of pixels in the field of view and multiplies the result by 100 to generate a percentage value. The average area of the target object was divided by viable cell counts to obtain average inclusion size per cell. Thresholds were kept uniform across individual experiments. Primary antibodies are listed in Table S3. Highly cross-adsorbed secondary antibodies were used at concentrations of 1.07 μg/mL (Jackson Laboratories) and are listed in Table S4.

For cold methanol fixation, primary hippocampal cultures plated on glass slides were exposed to ice-cold methanol (-20 ^o^C) for 15 minutes on DIV 4. Cells were then permeabilized and blocked using LI-COR Intercept Blocking Buffer PBS (927-70001, LI-COR) with 0.3 % Triton X-100 and immunostained as described above. After immunostaining, coverslips were applied using FluoroMount-G (0100-01, SouthernBiotech).

***SDS-PAGE and Immunoblotting:***

For *in vitro* studies, 1× Cell Lysis Buffer was prepared (9803, Cell Signaling Technologies) supplemented with 1 % protease inhibitor cocktail (P8340, Sigma-Aldrich), 1 mM phenylmethylsulfonylfluoride (PMSF; P7626, Sigma-Aldrich), 1 % protease inhibitor cocktail (In-house, 2 mM imidazole, 1 mM sodium fluoride, and 1 mM sodium orthovanadate), and 10 mM sodium fluoride (S7920, Sigma-Aldrich). Media were removed on DIV 12 and ice-cold cell lysis buffer was added to the cell culture wells. Plates were then incubated for 10 minutes on wet ice, and cells were scraped and sonicated (XL2020, Misonix Inc.). Protein content was determined by the bicinchoninic acid protein assay (23225, ThermoFisher Scientific) or NanoDrop One*^C^* (NanoDrop One C, ThermoFisher Scientific). Sample loading buffer (928-40004, LI-COR) containing 10 % fresh β-mercaptoethanol was added at a 1:3 dilution to each sample.

Ten micrograms of protein were loaded onto SDS-polyacrylamide gels and transferred to polyvinylidene difluoride (PVDF; Immobilon-FL PVDF, IPFL0010, Millipore) or nitrocellulose membranes (926-31092, LI-COR). Membranes were dried to minimize protein loss and PVDF membranes were rehydrated in methanol, whereas nitrocellulose membranes were rehydrated in water. When probing α-synuclein, 12 % acrylamide gels were subjected to SDS-PAGE for > 3 hours to improve spatial resolution. For all other proteins, 10 % acrylamide gels were used. After transfer, the dried and rehydrated membranes were also fixed in 4 % formalin (diluted from Formal-Fixx, 9990244, ThermoFisher Scientific) and 0.01 % glutaraldehyde in Tris-buffered saline for 15 minutes when probing α-synuclein.^9, 15, 16^

As a loading control, Total Protein staining was performed with the REVERT stain (926-11010, LI-COR). Briefly, membranes were exposed to REVERT diluted 1:5 in methanol for 1.5 minutes and then washed twice for 30 seconds with 6.7 % glacial acetic acid and 30 % methanol in water. The REVERT stain was fully removed by incubating in LI-COR REVERT Destaining solution (926-11013, LI-COR) for 11 minutes, prior to blocking with 100 % LI-COR Intercept (TBS) blocking solution (927-60003, LI-COR). Immunolabeling for the target proteins was carried out using primary and secondary antibodies, diluted in three parts LI-COR Intercept (TBS) Blocking Buffer (927-60003, LI-COR) in one part 10 mM Tris-buffered saline, with 0.1 % Tween-20. Primary and secondary immunoblotting antibodies are listed in Tables S3 and S4. Highly cross-adsorbed antibodies from Invitrogen and Jackson Laboratories were used for immunoblotting at 0.133 μg/mL and 0.05 μg/mL, respectively. The blots were imaged using the Odyssey Classic or the Odyssey M imager and quantified with LI-COR Image Studio.

Note that monomeric endogenous α-synuclein migrates through denaturing gels at ~17 kDa,^17^ whereas the denatured fibrils migrate at ~14 kDa, the predicted mass of recombinant α-synuclein monomers (Figure S4). As synthesized, the exogenous recombinant fibrils are not phosphorylated,^4, 18^ as confirmed on our immunoblots (arrows in Figure S4A). Changes in pSer129 as a function of total protein concentrations (Figure S4D-E), total protein concentrations in the cell lysates (Figure S4F), levels of denatured, exogenous α-synuclein (Figure S4G) and the levels of endogenous pan α-synuclein were slightly or not at all modified by **1** (Figure S4H)*.*

**Figure S2. Impact of Multitargeting Agents on α-Synucleinopathy in Vitro.** Primary rat hippocampal cultures were treated with **2** and preformed α-synuclein fibrils for 10 days *in vitro* before immunostaining for hyperphosphorylated α-synuclein (pSer129). The Hoechst reagent stained all nuclei. Quantification of (**A**) viable Hoechst^+^ cell counts per field of view under a 20× objective, (**B**) pSer129^+^ objects per Hoechst^+^ cell. Representative images of cells labeled with the Hoechst nuclear stain or immunolabeled for pSer129 α-synuclein are in panel **C**. Shown are the mean ± S.D. of *n* = 3 independent cultures, each run in duplicate or triplicate wells. Replicate wells from independent cultures were then averaged to yield a single number as the final statistical unit; this average number is plotted as a blue dot in the graphs. * *p* ≤ 0.0500, * * *p* ≤ 0.0100, two-way repeated measures (with matching for each independent culture) ANOVA/Bonferroni. Statistically significant effects of experimental Lewy body disease or fibril exposure were observed in panels **A-B** (*p* ≤ 0.0500), but the statistical impact of preformed fibrils in pairwise comparisons was not added, to avoid figure crowding. (**D**) Primary hippocampal cultures were treated with **1** (8 μM) or **2** (4 μM) for 10 days *in vitro*, along with preformed fibrils as indicated. Cultures were exposed to the nonionic surfactant and emulsifier Triton X-100 during fixation, for removal of soluble α-synuclein, and stained with the pan-nuclear marker Hoechst and antibodies against pSer129 α-synuclein. The scale bars in subpanels of C and D apply to all images in panels C and D, respectively.


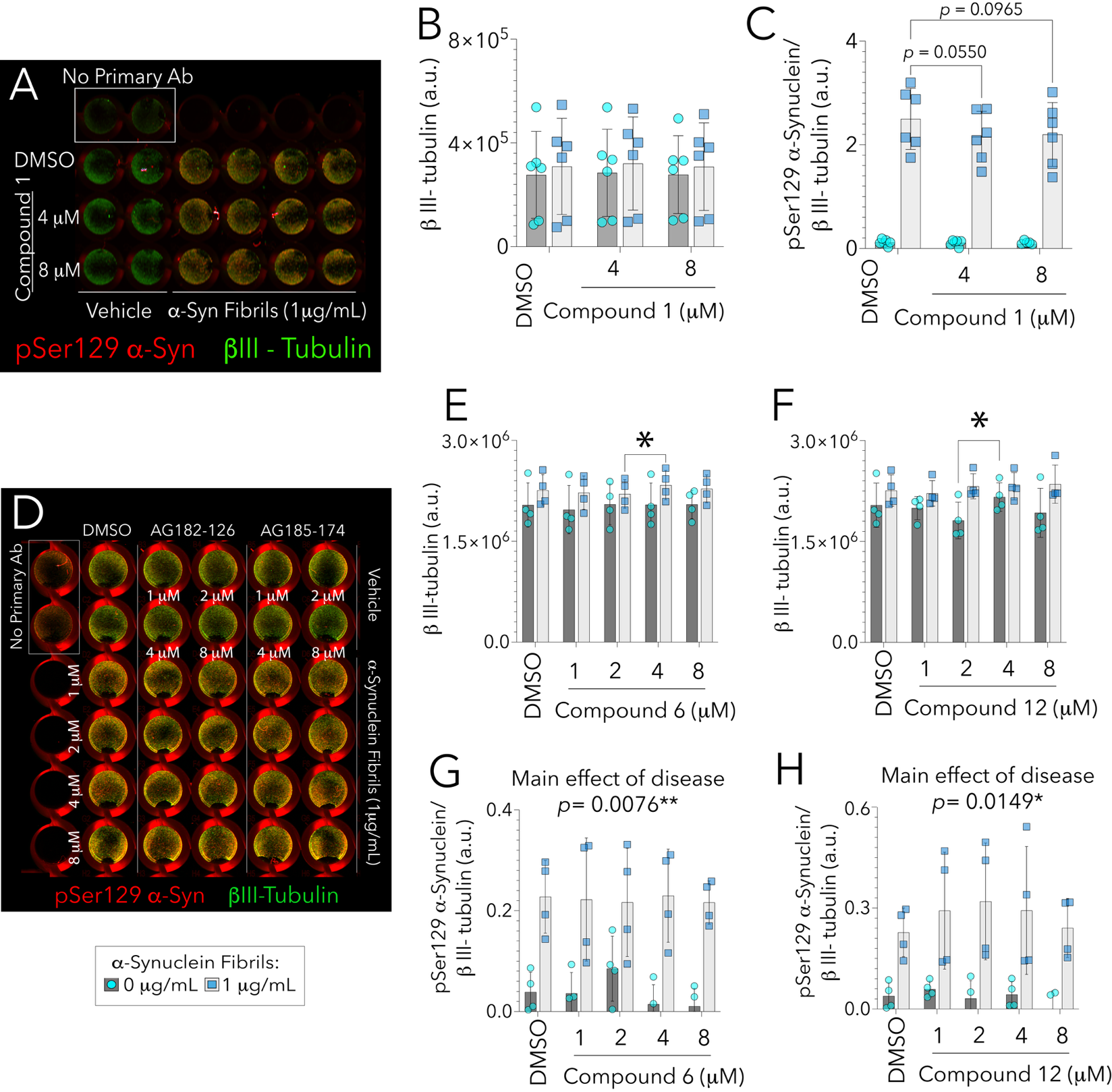


**Figure S3. Impact of Multitargeting Agents on α-Synucleinopathy in Vitro.** Primary hippocampal cultures were treated with preformed α-synuclein fibrils and **1**, **6** and **12** for 10 days *in vitro* before immunostaining for hyperphosphorylated α-synuclein (pSer129) as a marker of Lewy-like pathology, and the pan-neuronal marker βIII-tubulin as a viability marker (**A-H**). (**A, D**) Representative In-Cell Western analyses for pSer129 and βIII-tubulin. Raw fluorescence values for βIII-tubulin staining on the Odyssey Imager M are shown in **B, E** and **F**. pSer129 immunoreactive signal was expressed as a function of βIII-tubulin levels in **C, G** and **H**. Shown are the mean ± S.D. for *n* = 4 independent cultures, each run in single to quadruplicate wells for In-Cell Western analyses. * *p* ≤ 0.0500, * * *p* ≤ 0.0100, two-way repeated measures ANOVA/Bonferroni (with matching for each independent culture). Main effects of fibril exposure (*i.e.*, experimental Lewy body disease) are indicated above graphs. Statistically significant effects of experimental Lewy body disease (fibril exposure) were observed in pairwise comparisons (*p* ≤ 0.0500) but were not added, to avoid figure crowding.

**Figure S4. Impact of Multitargeting Agent 1 on α-Synucleinopathy in Vitro.** (**A-H**) Primary rat hippocampal cultures were treated in vitro for 10 days with preformed fibrils and **1** or respective vehicles. (**A**) Full-length representative immunoblots showing the loading control (Total Protein REVERT stain), pan α-synuclein, and hyperphosphorylated α-synuclein (pSer129). Note that the REVERT stain is chemically removed prior to primary antibody incubation. Arrows point to endogenous α-synuclein (monomeric α-synuclein migrates at ~17 kDa on denaturing gels) versus preformed (exogenous) and denatured fibrils. Please note that the band between 50 and 75 kDa is nonspecific binding from the 800 nm secondary antibody. Levels of (**B**-**C**) pSer129 α-synuclein as a fraction of pan α-synuclein, (**D**-**E**) pSer129 as a fraction of total protein concentrations, (**F**) total protein levels, (**G**) exogenously applied preformed fibrils, and (**H**) endogenous α-synuclein (*i.e*., excluding the synthetic fibrils). Shown are the mean ± S.D. of n = 5 independent cultures for all groups, each run in duplicate or triplicate wells. Replicate wells from independent cultures were averaged to yield a single number as the final statistical unit, plotted as a blue dot in the graphs. * *p* ≤ 0.0500, repeated measures (matching for each independent culture) two-way ANOVA/Bonferroni or a two-tailed paired *t* test on the fibril-treated groups alone (in **B-C** and **D-E**). Statistically significant effects of experimental Lewy body disease (fibril exposure) were observed in **B**, **D**, and **G** (*p* ≤ 0.0500), but the statistical impact of preformed fibrils in pairwise comparisons was not added to avoid figure crowding. (**I**-**L**) Primary hippocampal cultures were treated with vehicle, Compound **1** (8 μM) or the positive control nocodazole (50 nM) for 5 min prior to 15 minutes of cold methanol fixation. Cultures were immunostained for somatodendritic neuron marker MAP2 (green) and neuronal microtubule marker βIII-tubulin (red). Nuclei were labeled with the Hoechst reagent (blue). Note that microtubules become wavy after cold methanol exposure even under physiological conditions. Severe loss of axons and dendrites and beading of βIII-tubulin+ structures is observed after exposure to 0.05 μM nocodazole (arrows) but not after 8 μM Compound **1**. Scale bar for panels **I**-**J** is in **J**. Scale bar for panels **K-L** is in **L**. a.u. = arbitrary units.

***Impact of Compound 1 on microtubule stabilization markers:***

**
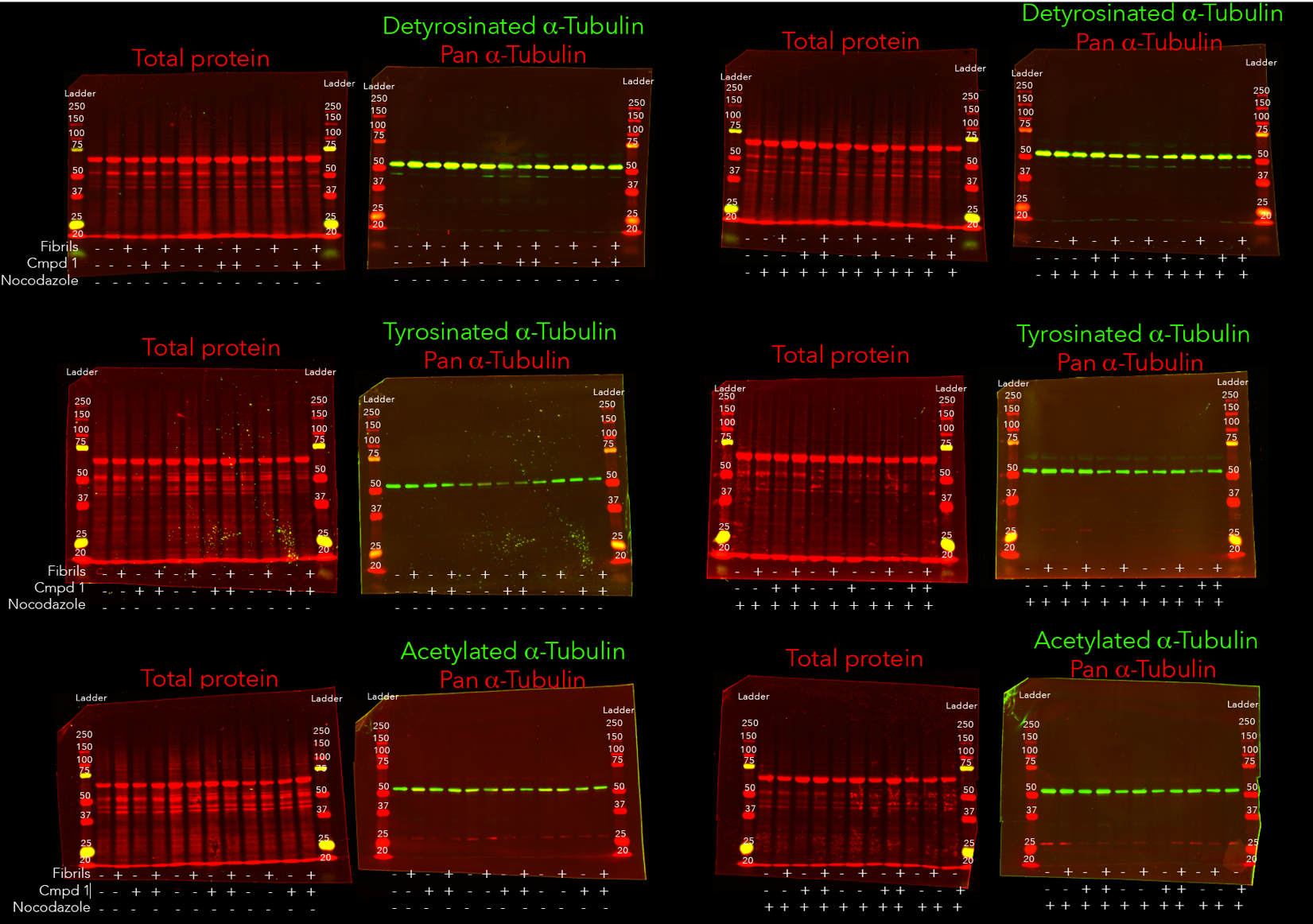
**

**Figure S5. Compound 1 subtly affects post-translational markers of microtubule stability.** Primary hippocampal cultures were treated for 10 days *in vitro* with preformed fibrils, Compound **1**, and nocodazole (or vehicle). Full-length representative immunoblots showing the loading control (Total Protein REVERT stain), pan α-tubulin, and detyrosinated, tyrosinated, or acetylated α-tubulin levels. Quantification of the levels of detyrosinated α-tubulin, tyrosinated α-tubulin, and acetylated α-tubulin as a fraction of pan α-tubulin are in Figure 9.

***Pharmacokinetic Analyses***

Pharmacokinetic analyses were completed for **2** and **1** at PharmaDirections by oral cassette administration (*n* = 9 male CD-1 mice per compound; Pharmaron Study Protocol PH-DMPK-PDS-21-001. Briefly, each compound was administered through oral routes, and plasma samplings were collected from 2 to 24 hours post-administration for analyses by high-performance liquid chromatography and mass spectrometry (Prominence (Degasser DGU-20A5R(C), Serial NO. L20705619752 IX; Liquid Chromatograph LC-30AD Serial NO. L20555611120 AE and L20555611116 AE; Communications Bus Module CBM-20A, Serial NO. L20235635001 CD, Auto SIL-20AC HT, Serial No. L20355305356 AE; Rack changer II, Serial No. L20585300757 SS; AB Sciex Triple Quan 5500 LC/MS/MS instrument (Serial NO. EF20711807)). A HALO 90A, C18 2.7 μm 2.1 x 50 mm USKL004340 column was used, and the mobile phase was 5 or 95 % acetonitrile in water with 0.1 % formic acid.

***References***

(1) Gangjee, A.; Zaware, N.; Raghavan, S.; Ihnat, M.; Shenoy, S.; Kisliuk, R. L. Single agents with designed combination chemotherapy potential: synthesis and evaluation of substituted pyrimido[4,5-b]indoles as receptor tyrosine kinase and thymidylate synthase inhibitors and as antitumor agents. *J Med Chem* **2010**, *53* (4), 1563-1578.

(2) Rohena, C. C.; Risinger, A. L.; Devambatla, R. K.; Dybdal-Hargreaves, N. F.; Kaul, R.; Choudhary, S.; Gangjee, A.; Mooberry, S. L. Janus Compounds, 5-Chloro-N(4)-methyl-N(4)-aryl-9H-pyrimido[4,5-b]indole-2,4-diamines, Cause Both Microtubule Depolymerizing and Stabilizing Effects. *Molecules* **2016**, *21* (12).

(3) *Schrödinger Release 2025-1: Maestro, Schrödinger, LLC, New York, NY, 2025.*; Maestro, Schrödinger, LLC, New York, NY, 2025.:

(4) Volpicelli-Daley, L. A.; Luk, K. C.; Lee, V. M. Addition of exogenous alpha-synuclein preformed fibrils to primary neuronal cultures to seed recruitment of endogenous alpha-synuclein to Lewy body and Lewy neurite-like aggregates. *Nature protocols* **2014**, *9* (9), 2135-2146.

(5) Polinski, N. K.; Volpicelli-Daley, L. A.; Sortwell, C. E.; Luk, K. C.; Cremades, N.; Gottler, L. M.; Froula, J.; Duffy, M. F.; Lee, V. M. Y.; Martinez, T. N.; et al. Best Practices for Generating and Using Alpha-Synuclein Pre-Formed Fibrils to Model Parkinson's Disease in Rodents. *J Parkinsons Dis* **2018**, *8* (2), 303-322.

(6) Bhatia, T. N.; Jamenis, A. S.; Abbas, M.; Clark, R. N.; Miner, K. M.; Chandwani, M. N.; Kim, R. E.; Hilinski, W.; O'Donnell, L. A.; Luk, K. C.; et al. A 14-day pulse of PLX5622 modifies alpha-synucleinopathy in preformed fibril-infused aged mice of both sexes. *Neurobiol Dis* **2023**, 106196.

(7) Miner, K. M.; Jamenis, A. S.; Bhatia, T. N.; Clark, R. N.; Rajasundaram, D.; Sauvaigo, S.; Mason, D. M.; Posimo, J. M.; Abraham, N.; DeMarco, B. A.; et al. alpha-synucleinopathy exerts sex-dimorphic effects on the multipurpose DNA repair/redox protein APE1 in mice and humans. *Prog Neurobiol* **2022**, *216*, 102307.

(8) Bhatia, T. N.; Clark, R. N.; Needham, P. G.; Miner, K. M.; Jamenis, A. S.; Eckhoff, E. A.; Abraham, N.; Hu, X.; Wipf, P.; Luk, K. C.; et al. Heat Shock Protein 70 as a Sex-Skewed Regulator of alpha-Synucleinopathy. *Neurotherapeutics* **2021**, *18* (4), 2541-2564.

(9) Miner, K. M.; Jamenis, A. S.; Bhatia, T. N.; Clark, R. N.; Abbas, M.; Luk, K. C.; Leak, R. K. The variance in phosphorylated, insoluble ⍺-synuclein in humans, rats, and mice is not mainly driven by biological sex. *Acta Neuropathol* **2023**, *146* (4), 651-654.

(10) Mason, D. M.; Wang, Y.; Bhatia, T. N.; Miner, K. M.; Trbojevic, S. A.; Stolz, J. F.; Luk, K. C.; Leak, R. K. The center of olfactory bulb-seeded alpha-synucleinopathy is the limbic system and the ensuing pathology is higher in male than in female mice. *Brain Pathol* **2019**, *29* (6), 741-770.

(11) Nouraei, N.; Mason, D. M.; Miner, K. M.; Carcella, M. A.; Bhatia, T. N.; Dumm, B. K.; Soni, D.; Johnson, D. A.; Luk, K. C.; Leak, R. K. Critical appraisal of pathology transmission in the alpha-synuclein fibril model of Lewy body disorders. *Exp Neurol* **2018**, *299* (Pt A), 172-196.

(12) Mason, D. M.; Nouraei, N.; Pant, D. B.; Miner, K. M.; Hutchison, D. F.; Luk, K. C.; Stolz, J. F.; Leak, R. K. Transmission of alpha-synucleinopathy from olfactory structures deep into the temporal lobe. *Mol Neurodegener* **2016**, *11* (1), 49.

(13) Posimo, J. M.; Unnithan, A. S.; Gleixner, A. M.; Choi, H. J.; Jiang, Y.; Pulugulla, S. H.; Leak, R. K. Viability assays for cells in culture. *J Vis Exp* **2014**, *83* (83), e50645.

(14) Eidet, J. R.; Pasovic, L.; Maria, R.; Jackson, C. J.; Utheim, T. P. Objective assessment of changes in nuclear morphology and cell distribution following induction of apoptosis. *Diagn Pathol* **2014**, *9*, 92.

(15) Lee, B. R.; Kamitani, T. Improved immunodetection of endogenous alpha-synuclein. *PLoS One* **2011**, *6* (8), e23939.

(16) Sasaki, A.; Arawaka, S.; Sato, H.; Kato, T. Sensitive western blotting for detection of endogenous Ser129-phosphorylated alpha-synuclein in intracellular and extracellular spaces. *Sci Rep* **2015**, *5*, 14211.

(17) Sharon, R.; Goldberg, M. S.; Bar-Josef, I.; Betensky, R. A.; Shen, J.; Selkoe, D. J. alpha-Synuclein occurs in lipid-rich high molecular weight complexes, binds fatty acids, and shows homology to the fatty acid-binding proteins. *Proc Natl Acad Sci U S A* **2001**, *98* (16), 9110-9115.

(18) Volpicelli-Daley, L. A.; Luk, K. C.; Patel, T. P.; Tanik, S. A.; Riddle, D. M.; Stieber, A.; Meaney, D. F.; Trojanowski, J. Q.; Lee, V. M. Exogenous alpha-synuclein fibrils induce Lewy body pathology leading to synaptic dysfunction and neuron death. *Neuron* **2011**, *72* (1), 57-71.
